# Increased expression and accumulation of GDF15 in IPF extracellular matrix contribute to fibrosis

**DOI:** 10.1172/jci.insight.153058

**Published:** 2022-08-22

**Authors:** Agata Radwanska, Christopher Travis Cottage, Antonio Piras, Catherine Overed-Sayer, Carina Sihlbom, Ramachandramouli Budida, Catherine Wrench, Jane Connor, Susan Monkley, Petra Hazon, Holger Schluter, Matthew J. Thomas, Cory M. Hogaboam, Lynne A. Murray

**Affiliations:** 1 Bioscience COPD/IPF, Research and Early Development, Respiratory and Immunology (R&I), BioPharmaceuticals R&D, AstraZeneca, Gothenburg, Sweden.; 2 Bioscience COPD/IPF, Research and Early Development, R&I, Biopharmaceuticals R&D, AstraZeneca, Gaithersburg, Maryland, USA.; 3 Bioscience In Vivo, Research and Early Development, R&I, BioPharmaceuticals R&D, AstraZeneca, Gothenburg, Sweden.; 4 Bioscience COPD/IPF, Research and Early Development, R&I, BioPharmaceuticals R&D, AstraZeneca, Cambridge, United Kingdom.; 5 Proteomics Core Facility of Sahlgrenska Academy, University of Gothenburg, Sweden.; 6 Translational Science and Experimental Medicine, Research and Early Development, R&I, BioPharmaceuticals R&D, AstraZeneca, Gothenburg, Sweden.; 7 Cedar-Sinai, Los Angeles, California, USA.

**Keywords:** Cell Biology, Pulmonology, Cytokines, Extracellular matrix, Fibrosis

## Abstract

Idiopathic pulmonary fibrosis (IPF) is a chronic disease of unmet medical need. It is characterized by formation of scar tissue leading to a progressive and irreversible decline in lung function. IPF is associated with repeated injury, which may alter the composition of the extracellular matrix (ECM). Here, we demonstrate that IPF patient–derived pulmonary ECM drives profibrotic response in normal human lung fibroblasts (NHLF) in a 3D spheroid assay. Next, we reveal distinct alterations in composition of the diseased ECM, identifying potentially novel associations with IPF. Growth differentiation factor 15 (GDF15) was identified among the most significantly upregulated proteins in the IPF lung–derived ECM. In vivo, GDF15 neutralization in a bleomycin-induced lung fibrosis model led to significantly less fibrosis. In vitro, recombinant GDF15 (rGDF15) stimulated α smooth muscle actin (αSMA) expression in NHLF, and this was mediated by the activin receptor-like kinase 5 (ALK5) receptor. Furthermore, in the presence of rGDF15, the migration of NHLF in collagen gel was reduced. In addition, we observed a cell type–dependent effect of GDF15 on the expression of cell senescence markers. Our data suggest that GDF15 mediates lung fibrosis through fibroblast activation and differentiation, implicating a potential direct role of this matrix-associated cytokine in promoting aberrant cell responses in disease.

## Introduction

Idiopathic pulmonary fibrosis (IPF) is an unrelenting and progressive lung disease. The available therapeutic options are limited due to a lack of their impact on mortality and, often, their intolerable side effects. The incidence of IPF is increasing likely due to improved diagnosis and awareness of disease.

Multiple mechanisms have been proposed to initiate and maintain lung fibrosis, with the central hallmark being heightened lung fibroblast activation (1). Fundamentally, normal healthy lung alveolar architecture is replaced with dense extracellular matrix (ECM), reducing the compliance of lung tissue and impacting gas exchange (2). Stiff IPF matrix has been shown to directly promote fibroblast activation and differentiation to myofibroblasts (3). The fibrotic environment is hypothesized to perpetuate the damage as the key profibrotic growth factor linked to IPF, TGF-β, is stored in a latent form in the matrix (4–6), waiting to be activated by a number of potential pathways. Active TGF-β then acts on fibroblasts to further enhance collagen deposition and differentiation to myofibroblasts (7). The mechanisms by which matrix promotes fibrotic responses in mesenchymal cells have been linked to various pathways, including integrins as key mechanosensors and ion channels as mechanosensitive receptors (8).

TGF-β1 is the prototypic growth factor that activates lung fibroblasts and induces ECM gene and protein expression (7). Other members of the TGF-β family have also been implicated in regulation of fibroblast function. Growth differentiation factor 15 (GDF15) is distantly related to other TGF-β family proteins, sharing 15%–29% amino acid similarity (9). It is secreted as a precursor and undergoes proteolytic cleavage to generate mature GDF15 that circulates as 24.5 kDa homodimer (10, 11). The only known receptor for GDF15 is found exclusively in the brain (12); therefore, how this cytokine may be functioning peripherally is currently unknown.

GDF15 is widely but weakly expressed in a nondisease state but is upregulated upon tissue injury and inflammation. Its overexpression is often associated with disease progression in cancer and diabetes, as well as cardiovascular and inflammatory diseases (13–17). This cytokine is known to be a key component of the senescence-associated secretory phenotype (SASP) (18, 19). In chronic obstructive pulmonary disease (COPD), high levels of plasma GDF15 have been associated with increased exacerbations (20). Moreover, it has been reported that GDF15 might promote airway epithelial senescence upon cigarette smoke exposure (21).

We profiled ECM from IPF and healthy lung tissue and demonstrated that IPF but not healthy matrices elicited a profibrotic response in lung fibroblasts and that the IPF matrix contained a number of differentially expressed proteins (compared with healthy lung), including the TGF-β family member GDF15. In vivo, when GDF15 was neutralized from 14 days and onward after initiation of bleomycin-induced lung fibrosis, we observed significantly less lung fibrosis, most notably due to decreased collagen deposition in fibrotic tissue areas. Mechanistically, the impact of GDF15 on cell senescence was cell type dependent, indicating that GDF15 plays a protective role in lung fibroblasts (preventing senescence), but it might be involved in promoting senescence in small airway epithelial cells (SAEC). We further demonstrated that recombinant GDF15 (rGDF15) directly induces collagen and α smooth muscle actin (αSMA) expression in lung fibroblasts. In addition, rGDF15-induced myofibroblasts were characterized by decreased migration capacity in 3D collagen gel–based migration assay. Taken together, our data suggest that GDF15 is directly linked to lung fibrosis and may play a role in cell senescence.

## Results

### Diseased lung ECM promotes fibroblast to myofibroblast transition.

**Figure 1:**
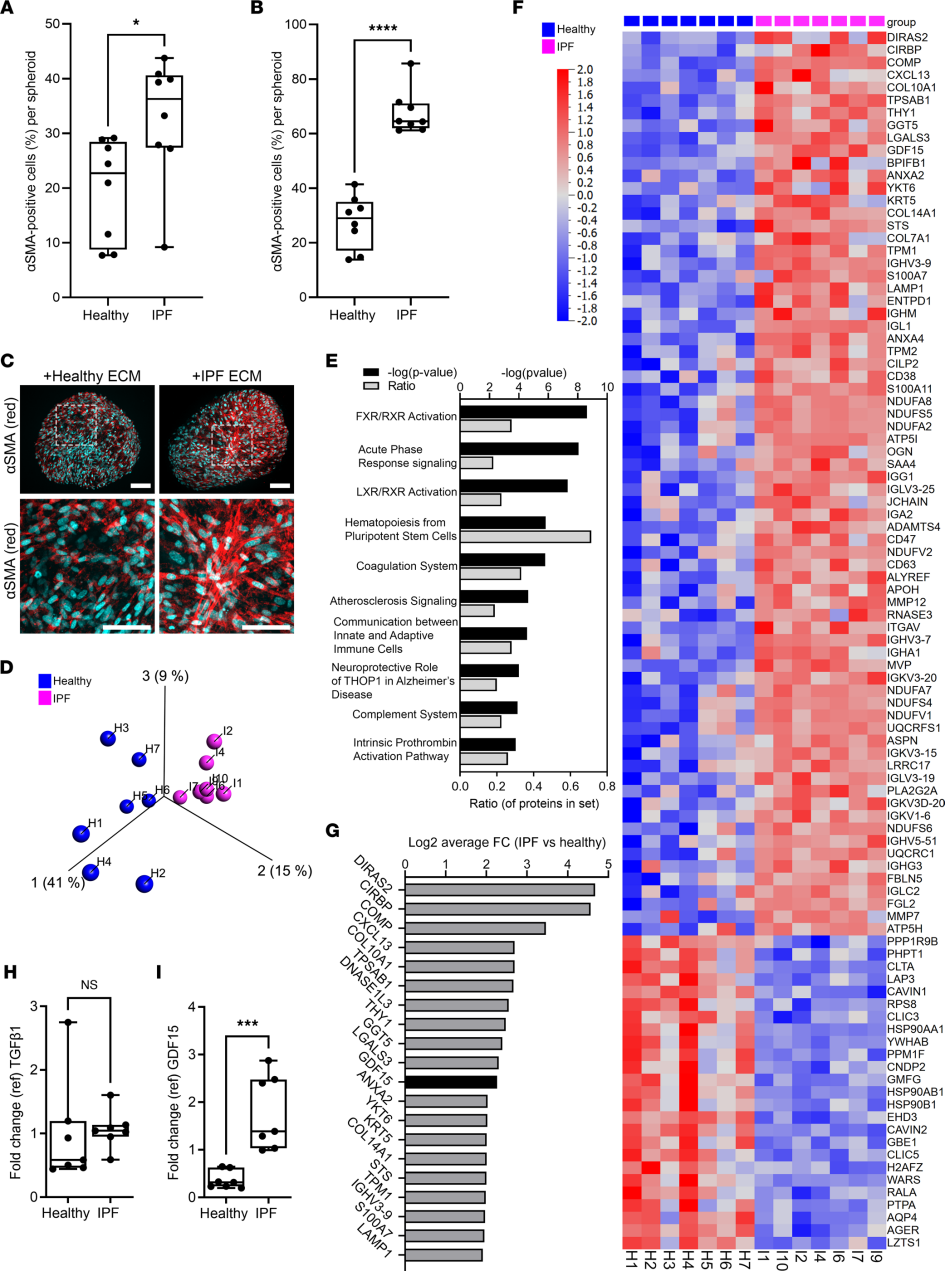
Diseased lung ECM promotes fibroblast-to-myofibroblast transition. (**A** and **B**) Decellularized and weight-normalized ECM from healthy or IPF lung (*n* = 8 donors) was cocultured with NHLF using 3D spheroid hanging drop system, and αSMA and nuclei were immunofluorescence labeled. Image analysis–based αSMA quantification was performed using ImageXpress Micro system, and the percentage of αSMA^+^ cells was calculated in each spheroid (>14 spheroids per patient) using MetaXpress High Content Image Analysis Software at day 4 (**A**) and day 8 (**B**) of culture. The data are presented as mean ± SD, with dots representing single patients, and they were statistically analyzed with 2-tailed Mann Whitney *U* test in comparison with healthy subjects; *P < 0.05 and ****P < 0.0001. (**C**) Representative immunofluorescence staining (5 experiments conducted) of αSMA (red) and nuclei (blue) in 3D spheroids, day 8. Magnified area is marked with dotted line. Scale bar: 100 μm. (**D**) PCA plot representing the data from proteomic analysis of decellularized ECM derived from healthy lung (blue; *n* = 7 donors) and IPF lung (pink; *n* = 7 donors). (**E**) IPA analysis of the top 10 significantly (–log[*P* value] > 1.30) enriched canonical pathways based on differentially expressed proteins in IPF ECM compared with healthy ECM. The data are presented as IPA –log(*P* value), and the ratio of proteins is represented in each pathway. (**F**) Heatmap comparison of significantly altered proteins (FC > 2.8 and FC < 0.36, *q* < 0.05, statistically analyzed with unpaired Student’s *t* test), identified in the proteome of IPF (pink) and healthy (blue) ECM from patient lung-derived samples. (**G**) The top-ranked proteins identified in IPF ECM plotted based on abundance and compared with healthy ECM. The data are expressed as log_2_FC. (**H** and **I**) Box-and-whisker plots representing FC over internal control (ref) for TGF-β1 (**H**) and GDF15 (**I**) identified in the proteome of healthy and IPF lung ECM (*n* = 7, dots represent single patients). Statistically analyzed with Mann Whitney *U* test; ***P < 0.001.

In order to assess the impact of pulmonary ECM on fibroblast to myofibroblast transition, normal human lung fibroblasts (NHLF) were cultured with cell-free ECM generated from patient-derived healthy lung tissue or IPF lung tissue (Supplemental Table 1; supplemental material available online with this article; undefinedDS1) in a 3D spheroid assay. Imaging of αSMA^+^ cells within the spheroids 4 days or 8 days after culture determined that IPF matrix mediated greater profibrotic responses in fibroblasts, compared with a healthy tissue–derived matrix (Figure 1, A–C, and Supplemental Figure 1). Proteomic analysis of the lung matrix indicated distinct separation of healthy- and IPF-derived samples following principal component analysis (PCA) with variance filtering, retaining 1070 variables out of 5356 initially detected (Figure 1D). Principal component 1, which accounted for 41% of the variability among samples, drew a clear separation between samples from healthy and IPF-patient derived matrices (Figure 1D). Principal component 2 accounted for an additional 15% variability between samples, and principal component 3 accounted for another 9% of variability. Ingenuity Pathway Analysis (IPA) of proteomic data suggested that IPF matrix showed enrichment of pathways associated with inflammation, such as acute phase response signaling, complement system, and communication between innate and adaptive immune cells (Figure 1E and Supplemental Table 2). Heatmap profiling of differentially expressed proteins identified 97 peptides whose expression was significantly different (*q* < 0.05) between the IPF and healthy group, of which 72 proteins were more abundant (average fold change [FC] > 2.8) and 25 proteins were less abundant (average FC < 0.36) in IPF ECM compared with healthy ECM (Figure 1F). Among the top-ranked proteins with increased expression in IPF matrix compared with healthy lung matrix, there is a number of collagen proteins (COL10, COL14, COL7), immune response-related proteins (CXCL13, LGALS3, IGHV3-9), and GDF15, a TGF-β family member (Figure 1, G and H). However, no statistically significant difference in TGF-β1 level was detected in the ECM of patients with IPF and healthy individuals (Figure 1I).

### GDF15 expression is increased in IPF lung and is localized in histopathological lesions.

**Figure 2:**
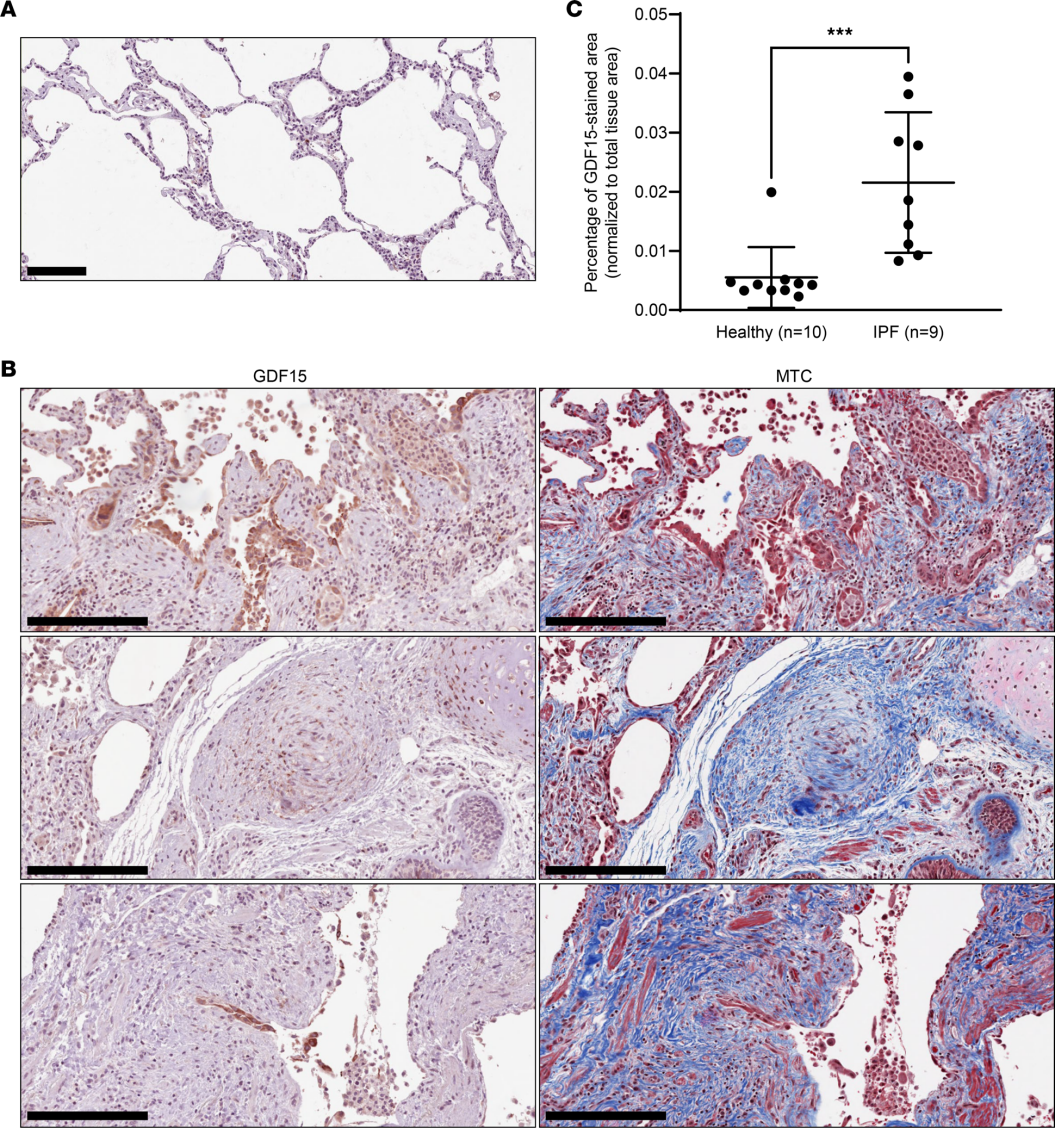
GDF15 expression increases in IPF lung and in histopathological lesions. (**A**) Representative IHC GDF15 staining of human lung tissue derived from healthy individual (*n* = 10). (**B**) Representative IHC GDF15 staining of human lung tissue from patients with IPF (*n* = 9) and corresponding MTC-stained sections. Arrows indicate GDF1^+^ cells in fibroblast foci (**B**, middle panel) and hyperplastic epithelium of small airways (**B**, bottom panel) in lung peripheral tissue. Scale bars: 100 μm (**A**) and 250 μm (**B**). (**C**) GDF15 expression in whole sections from healthy (*n* = 10) and IPF (*n* = 9) lung biopsies was quantified using Visiopharm software. Data are shown as mean ± SD. ***P = 0.0004, using 2-tailed unpaired Mann-Whitney *U* test.

**Figure 3:**
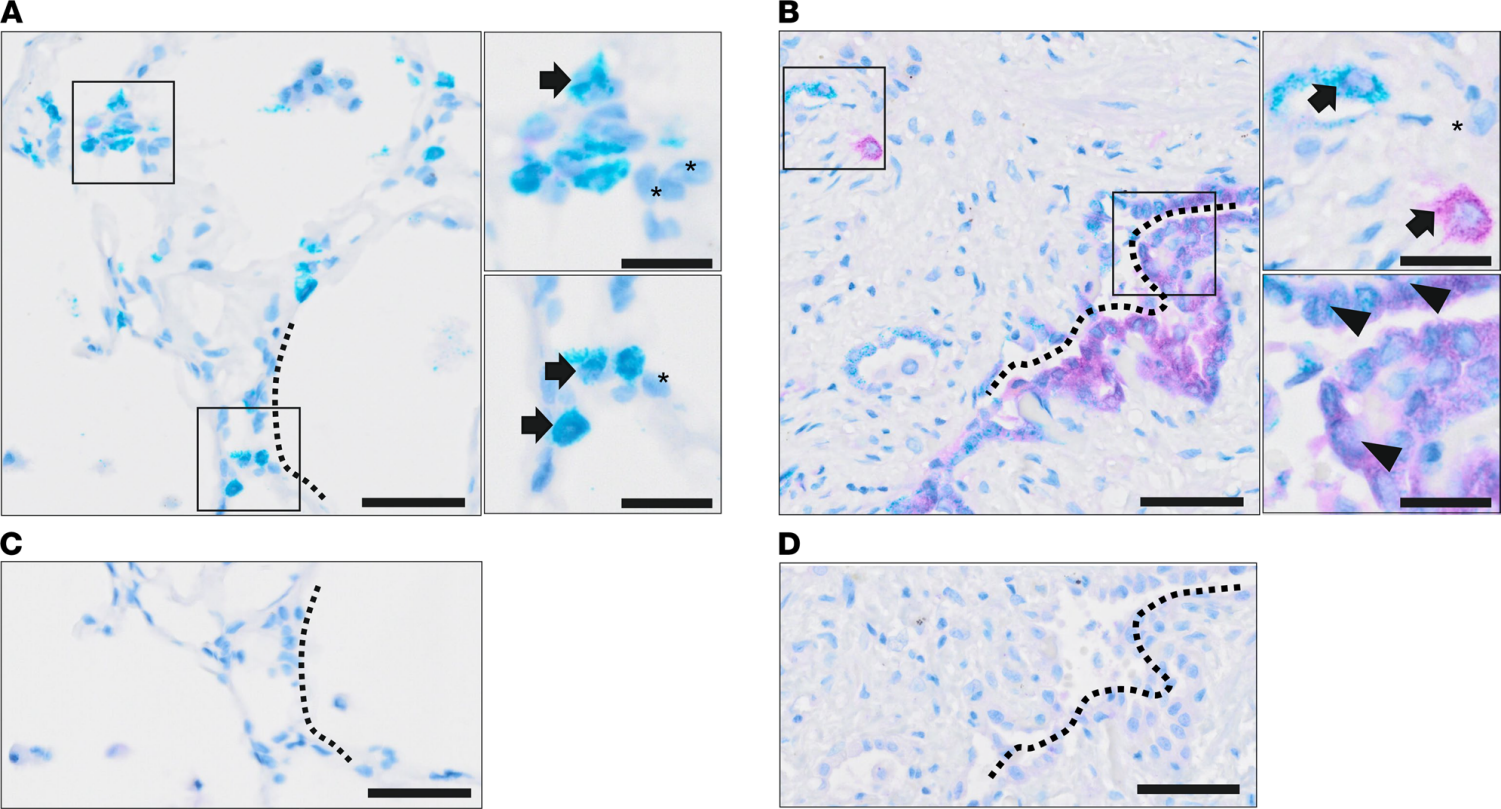
GDF15 expression in ATII cells. (**A** and **B**) Representative double IHC staining of GDF15 (purple) and PSPC (teal) in human lung tissue derived from healthy individual (*n* = 10) (**A**) and from patients with IPF (*n* = 9) (**B**). Magnified areas are marked with boxes. Arrowheads indicate cells coexpressing GDF15 and PSPC, arrows show positive cells for a single staining (GDF15 or PSPC), and asterisks depict negative cells. Scale bars: 100 μm and 20 μm (magnified areas). (**C** and **D**) Sequential section was used as a negative control for healthy (**C**) and IPF (**D**) samples in matched areas, as indicated by dashed lines.

To determine the relevance of GDF15 to the disease and localize the expression of GDF15 in lung tissue, we performed IHC staining in lung sections from patients diagnosed with IPF (Supplemental Table 1) and from healthy donors. We observed an increase in GDF15 staining in IPF sections compared with the healthy donors (Figure 2, A–C), confirming our proteomic analyses. Moreover, GDF15 expression was detected in fibroblastic foci and epithelial hyperplastic cells in patients with IPF (Figure 2B). In contrast, GDF15 was rarely detected in interstitial areas of normal healthy lung (Figure 2, A and C). Next, we performed costaining of GDF15 with alveolar epithelial type II (ATII) cells marker ProSurfactant protein C (PSPC) in human IPF and healthy lungs, confirming a colocalization of GDF15 in PSPC^+^ cells (Figure 3, A–D).

### Neutralization of GDF15 attenuates bleomycin-induced collagen deposition.

**Figure 4:**
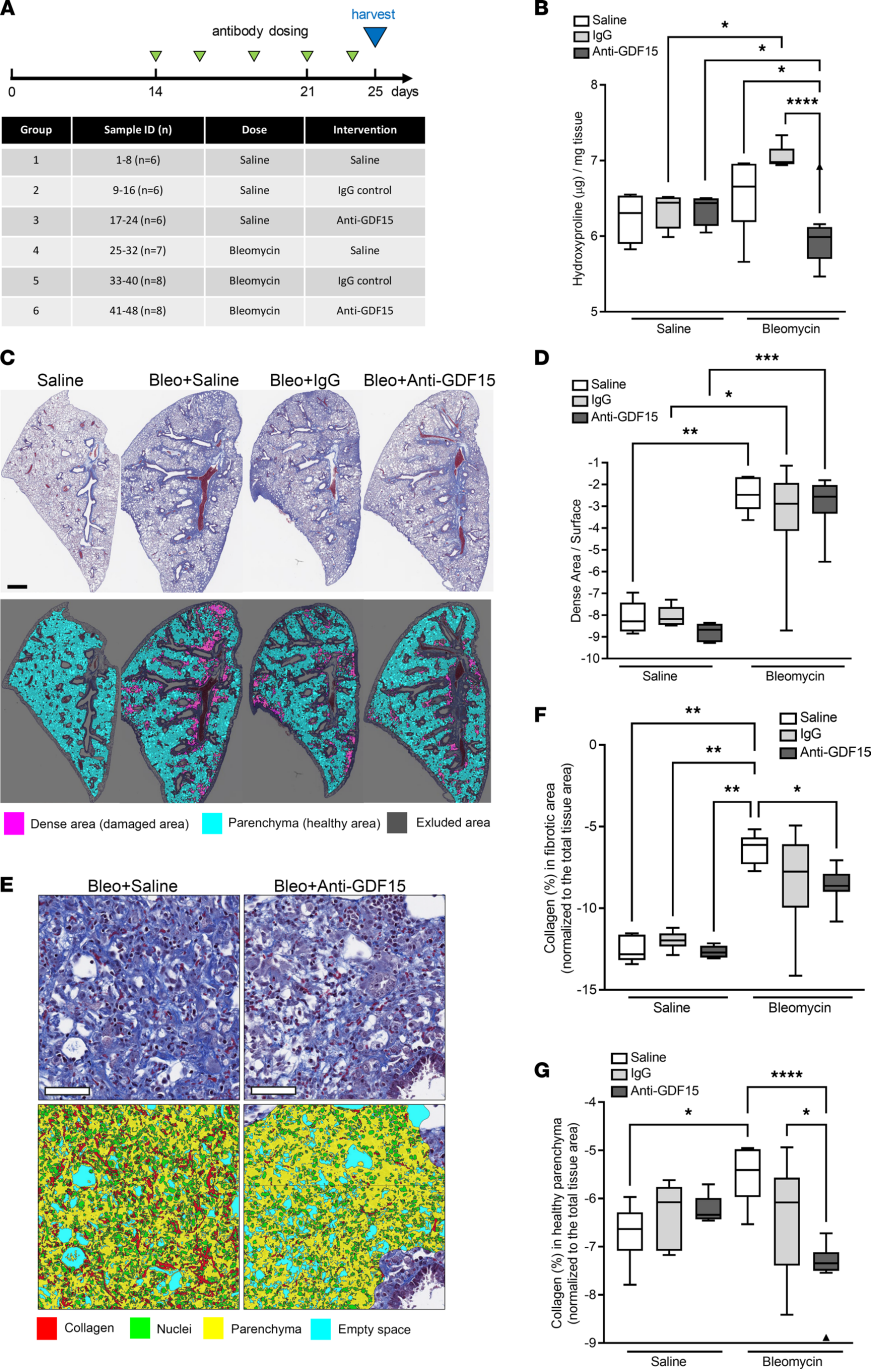
Neutralization of GDF15 attenuates bleomycin-induced collagen deposition. (**A**) Schematic representation of experimental procedure for GDF15 mAb administration in bleomycin-induced fibrosis model. (**B**) Lung tissue hydroxyproline content was measured in differently treated animals. (**C**) Representative MTC-stained sections (top panel; collagen in blue, nuclei in red) and the respective digital reconstructed images (bottom panel) in Visiopharm software correspond to the lung sections from differently treated groups (*n* = 6–8 animals per group). Healthy parenchyma (cyan) and dense damaged area (purple) are marked. (**D**) Imaged-based quantification of dense damaged area in differently treated groups. (**E**) High magnification of dense MTC-stained damaged area (top panel) in bleomycin-treated (bleo) lungs with and without anti-GDF15 antibody, and corresponding classifiers obtained by Visiopharm software (bottom panel; collagen marked in red). (**F** and **G**) Quantification of collagen (after MTC staining) in dense damaged area and in healthy parenchymal area in murine lungs from differently treated groups. Data are presented as box-and-whisker plots. *n* = 6–8; *P < 0.05, **P < 0.01, ***P < 0.001, and ****P < 0.0001 (analyzed using multiple Kruskal-Wallis test). Scale bars: 1 mm (**B**) and 50 μm (**E**).

It has been previously reported that GDF15 expression increases in bleomycin-induced fibrosis (22, 23). In order to determine the effect of GDF15 neutralization in vivo, we employed a bleomycin-mediated lung fibrosis model in mice and administered anti-GDF15 monoclonal antibody 14 days after initiation of injury (Figure 4A). A previous study has reported that *Gdf15^–/–^* mice are not protected against fibrosis in an intratracheal bleomycin model (22); therefore, we sought to determine if therapeutic dosing of a specific anti-GDF15 mAb would confer antifibrotic activity. Here, we determined that mice treated with anti-GDF15 mAb had significantly lower lung fibrosis, as measured by total lung hydroxyproline, a major component of collagen (Figure 4B).

Next we assessed the extent of histopathological lesions and fibrotic collagen deposition using sections stained for Masson’s trichrome (MTC). Automatic histopathological analysis of dense damage area (which is a sum of different histomorphological changes) demonstrated increased fibrotic changes in bleomycin-treated mice compared with the control group (Figure 4, C and D), but administration of GDF15-neutralizing antibody didn’t have any significant effect on the extent of dense damage area formation. However, automatic quantification of collagen content confirmed that GDF15 neutralization in bleomycin-treated mice resulted in significant reduction of collagen deposition in the whole lung sections in comparison with animals receiving only saline, both in fibrotic dense damaged area and in healthy-like nonfibrotic parenchyma (Figure 4, E–G). Altogether, these data show that treatment with anti-GDF15 antibody significantly decreased the level of collagen in the bleomycin model.

**Figure 5:**
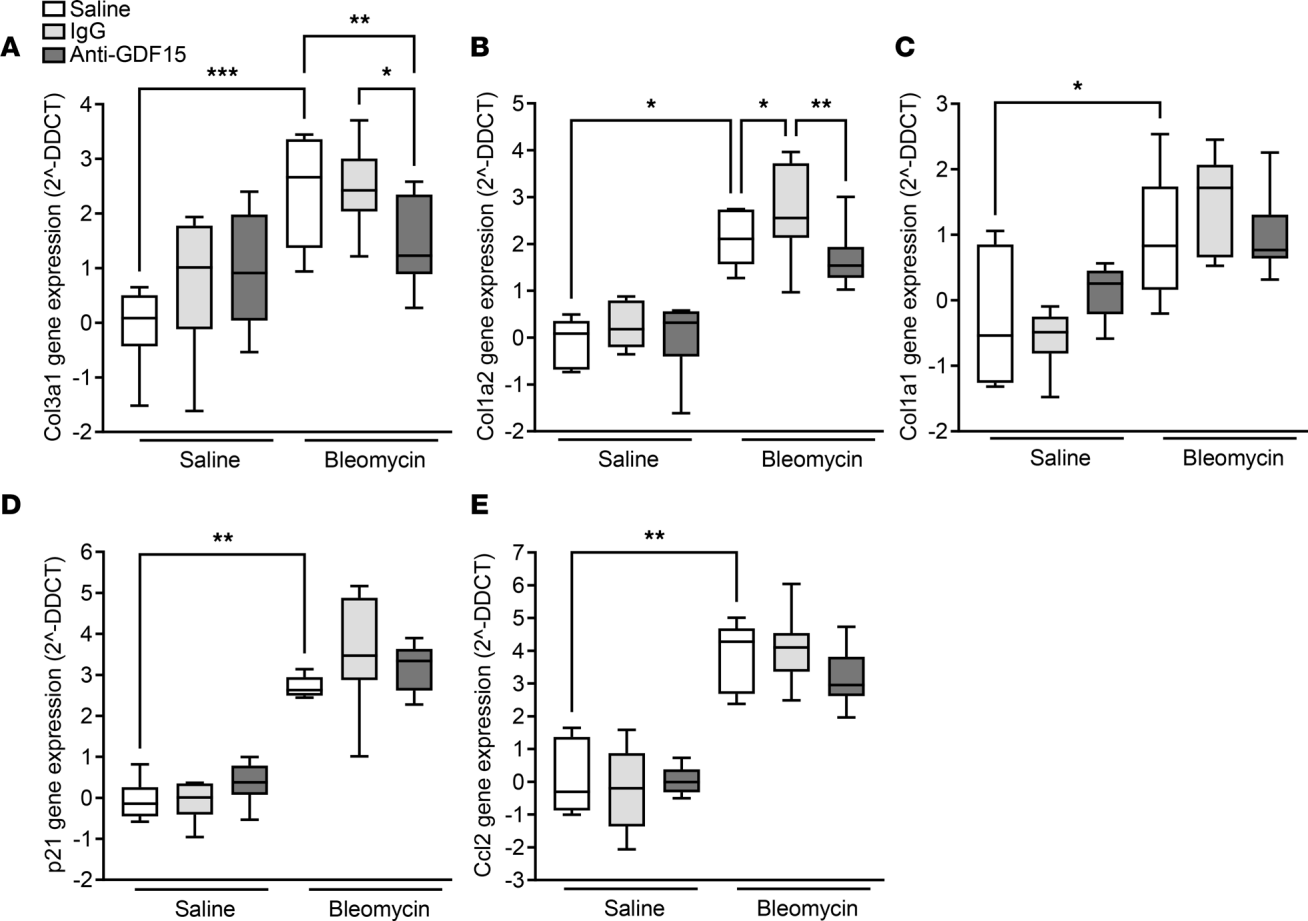
GDF15 neutralization attenuates bleomycin-induced fibrotic gene expression. (**A**–**E**) qPCR analysis of profibrotic gene expression in the lung of bleomycin-treated mice. The impact of GDF15 neutralization on the bleomycin-induced expression of *Col3a1* (**A**), *Col1a2* (**B**), *Col1a1* (**C**), *p21* (**D**), and *Ccl2* (**E**) genes. The expression of the genes of interest was calculated after the normalization to *18S* housekeeping gene expression, and the data are presented as 2^–ΔΔCT^, corresponding to the fold change in gene expression above saline/saline control mouse group. Data are presented as box-and-whisker plots. *n* = 6–8; *P < 0.05, **P < 0.01, and ***P < 0.001 (analyzed using multiple comparison Kruskal-Wallis test).

Assessment of ECM gene expression identified a significant reduction in bleomycin-induced *Col3a1* (Figure 5A) in the lungs of anti-GDF15–treated animals, but no significant impact on *Col1* (Figure 5, B and C) was detected. However, *Col1a2* gene expression was significantly reduced in the lungs of anti-GDF15–treated animals when compared with IgG-treated animals (Figure 5B). GDF15 was previously linked to the SASP; thus, we investigated the impact of GDF15 neutralization in bleomycin-treated mice on the expression of well-recognized senescence markers, such as *p21 (Cdkn1a)* and *Ccl2* (24). GDF15 neutralization did not have any significant impact of *p21* and *Ccl2* expression (Figure 5, D and E).

### GDF15 promotes profibrotic responses in lung fibroblasts.

**Figure 6:**
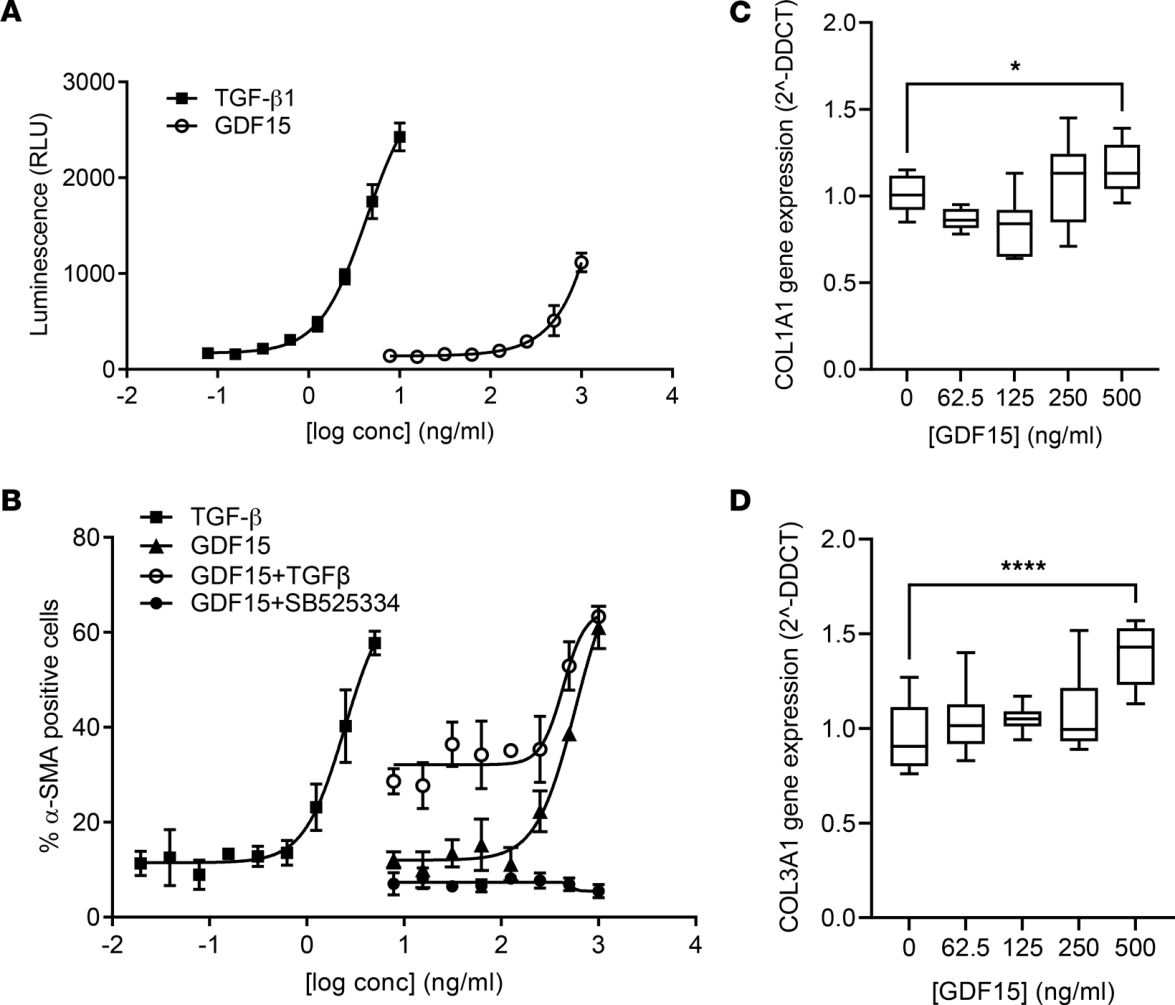
GDF15 promotes profibrotic responses in lung fibroblasts. (**A**) TMLC reporter cells were stimulated with rGDF15 or TGF-β for 24 hours, and luminescence corresponding to PAI1 receptor activity was measured; *n* = 3 biological replicates. (**B**) NHLF were incubated for 48 hours with rGDF15 alone, and/or with 0.5 ng/mL of TGF-β, or with SB525334 inhibitor. Next, αSMA and nuclei were immunofluorescence labeled, and the percentage of αSMA^+^ cells was quantified using MetaXpress High Content Image Analysis Software. Data are expressed as mean ± SD of *n* = 3 biological replicates. (**C** and **D**) The expression of *COL1A1* (**C**) and *COL3A1* (**D**) in rGDF15-stimulated NHLF was calculated after the normalization to *GAPDH* housekeeping gene expression and compared with untreated cells, shown as 2^–ΔΔCT^ (fold change) and presented using box-and-whisker plots, with the line in the middle plotted at the median. *P < 0.05 and ****P < 0.000 by 1-way ANOVA; *n* = 4 biological replicates.

In order to determine the potential mechanism through which GDF15 is promoting fibrosis, we profiled the recombinant protein on a number of fibrosis-related endpoints in NHLF. TGF-β stimulation of primary NHLF resulted in increased *GDF15* gene expression, but only when high concentrations of TGF-β were used, and rGDF15 had no direct effect on inducing *TGFB1* gene expression in NHLF (Supplemental Figure 2). We observed that GDF15 signals via the TGF-β pathway, as measured in the transformed mink lung epithelial cells (TMLC) expressing PAI1 promoter luciferase (25), albeit much less potently that TGF-β1 alone (Figure 6A). We then cultured NHLF with rGDF15 for 48 hours and measured the percentage of the cells expressing αSMA, as a surrogate for fibroblast-myofibroblast transition (FMT). Here, rGDF15 induced an upregulation of αSMA expression, and this activity was additive to TGF-β alone and was completely abolished with activin receptor-like kinase 5 (ALK5) inhibition using a small molecule inhibitor SB525334 (Figure 6B). To further assess the impact of GDF15 on fibroblast synthetic capacity, we stimulated lung fibroblasts for 48 hours with rGDF15 and observed a moderate increase in *COL1A1* (Figure 6C) and *COL3A1* (Figure 6D) genes expression.

### Stimulation with rGDF15 does not affect cell proliferation but leads to a decreased migration of NHLF.

**Figure 7:**
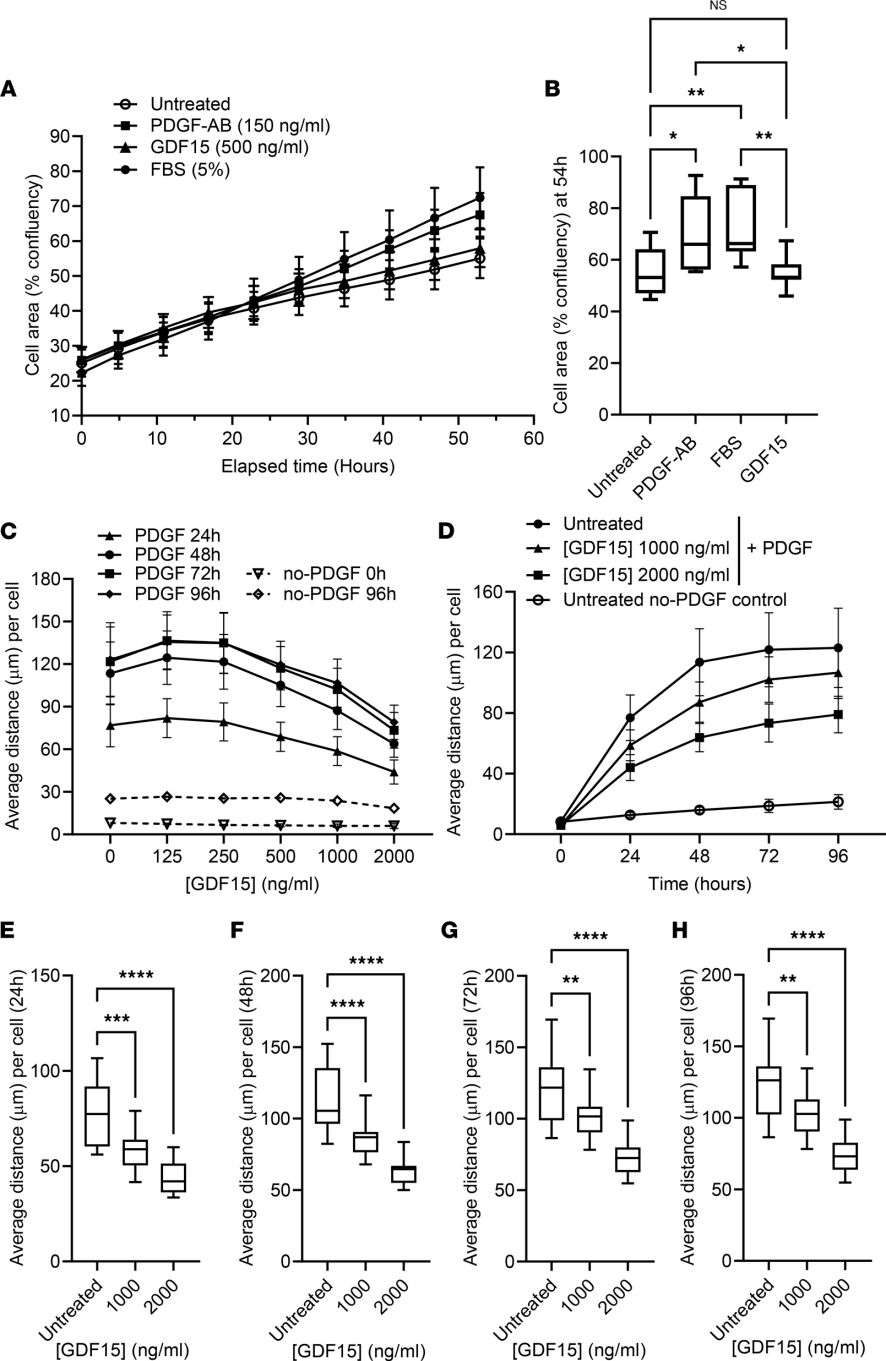
Stimulation with rGDF15 does not affect cell proliferation but leads to a decreased migration of NHLF. (**A** and **B**) For assessment of cell proliferation rate, 1.6 × 10^3^ NHLF per well were plated in the presence of rGDF15, PDGF-AB, or 5% FBS, and the cell confluency (%) was calculated over 55 hours of incubation using Incucyte system. Data are expressed as mean ± SD value over time (**A**) or using box-and-whisker plot (with the line in the middle plotted at the median) at 54 hours (**B**), statistically analyzed using multiple Kruskal-Wallis test. *P < 0.05, **P < 0.01; *n* = 3 biological replicates. (**C**) NHLF were embedded in collagen gel with or without rGDF15. After 6 hours of incubation, PDGF-BB was added to form a stable gradient, and migration of NHLF was tracked using confocal live-imaging system Yokogawa CV7000 for 96 hours. Average migration distance per cell was calculated using Columbus software. (**D**) PDGF-induced migration of NHLF was compared with no-PDGF, untreated control. (**E**–**H**) The data were statistically analyzed at 24 (**E**), 48 (**F**), 72 (**G**) and 96 (**H**) hours of migration toward PDGF-BB with multiple 1-way ANOVA test. **P < 0.01, ***P < 0.001 and ****P < 0.0001; *n* = 3 biological replicates. Data are expressed as mean ± SD (**C** and **D**) or as box-and-whisker plots (**E**–**H**), with the line in the middle plotted at the median.

Along with excess collagen deposition, fibroproliferation is a hallmark of IPF (1). Using an in vitro time course model, we determined that rGDF15 did not directly influence NHLF proliferation in comparison with unstimulated cells, in contrast to PDGF-AB and FBS (Figure 7A). The data were statistically analyzed compared with unstimulated cells and PDGF-AB or FBS at 54 hours (Figure 7B), demonstrating no significant effect of rGDF15 on NHLF proliferation. Both PDGF-AB and 5% FBS stimulated the cell proliferation, and these effects were statistically significant compared with unstimulated cells or rGDF15.

In order to characterize further the GDF15-activated fibroblasts, we performed the migration assays in collagen gel, measuring average migration distance toward PDGF-BB at 24–96 hours (Figure 7, C–H). Stimulation with rGDF15 resulted in a dose-dependent decrease in average migration distance of NHLF toward PDGF-BB (Figure 7C), and the most pronounced effect was observed in the presence of the highest doses (1000 and 2000 ng/mL) of rGDF15 (Figure 7, C–H). Additionally, we have assessed the effect of rGDF15 on the spontaneous migration of NHLF without addition of PDGF-BB (Supplemental Figure 3), demonstrating that the presence of the highest dose (2 µg/mL) of rGDF15 led to the decreased cellular migration at 24 and 48 hours (Supplemental Figure 3, B–D).

### The impact of GDF15 on cell senescence is cell type dependent.

**Figure 8:**
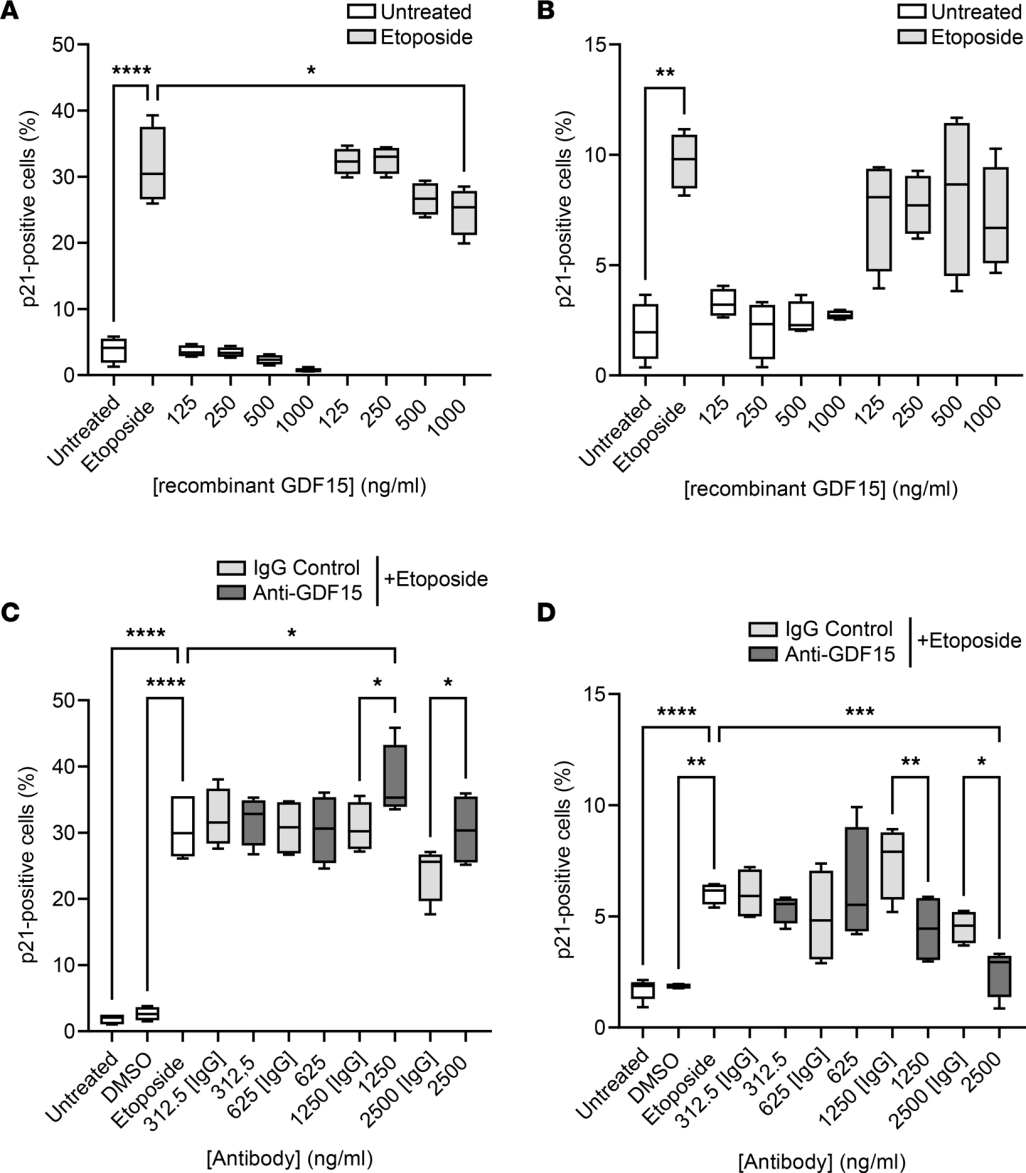
The effect of GDF15 on cellular senescence. (**A**–**D**) Cells were stimulated with etoposide (3 μM) or DMSO vehicle control for 24 hours. The percentage of P21^+^ NHLF (**A** and **C**) or SAEC (**B** and **D**) was calculated using immunolocalization imaging and MetaXpress High Content Image Analysis Software after preincubation with recombinant GDF15 (**A** and **B**) and after preincubation with GDF15-neutralizing antibody (**C** and **D**). Data (*n* = 4 biological replicates) are presented using box-and-whisker plots; the line in the middle plotted at the median. Statistically analyzed with multiple ANOVA analysis with Tukey’s post hoc test. *P < 0.05, **P < 0.01, ***P < 0.001, and ****P < 0.0001.

IPF is linked to both fibroblast senescence and epithelial cell senescence (26). To more comprehensively explore the potential of GDF15 at contributing to the cellular senescence, we assessed both the impact of recombinant protein and GDF15 neutralization in etoposide-induced senescence assays. In both the NHLF and SAEC assays, *GDF15* gene expression was upregulated by etoposide (Supplemental Figure 4), as was the expression of senescence marker p21 (Figure 8 and Supplemental Figure 5). In the presence of the highest dose (1000 ng/mL) of rGDF15, we observed a modest decrease in the percentage of etoposide-induced P21^+^ NHLF (Figure 8A), but there was no significant effect on *p21* (*CDKN1A*) gene expression in these cells (Supplemental Figure 5A). In the presence of GDF15-neutralizing antibody, the percentage of etoposide-induced P21^+^ NHLF increased compared with etoposide (this effect was statistically significant for 1250 ng/mL) and etoposide-treated IgG control (Figure 8C). This was reflected by a trend toward an increase of the *p21* (*CDKN1A*) gene expression in these cells (compared with etoposide-treated IgG control), but this effect was not statistically significant (Supplemental Figure 5C). The preincubation with rGDF15 had no impact on p21 level in SAEC (Figure 8B and Supplemental Figure 5B). However, in the presence of the highest doses (1250 and 2500 ng/mL) of the GDF15 neutralizing antibody, the percentage of SAEC expressing etoposide-induced P21 was decreased (Figure 8D), but no significant change in *p21* (*CDKN1A*) gene expression was detected (Supplemental Figure 5D). To confirm that further, we performed additional senescence assay, using Cellular Senescence Detection Kit–SPiDER-βGal. We detected the expression of senescence-associated β-galactosidase (SA–β-gal) in SAEC upon etoposide treatment (Supplemental Figure 6). This effect was attenuated in the presence of the highest dose (2500 ng/mL) of GDF15 neutralizing antibody compared with the etoposide-treated cells with or without IgG control (Supplemental Figure 6).

## Discussion

We have shown that GDF15 is one of the most abundant proteins in the IPF lung, expressed in multiple regions within the lung. Blocking GDF15 in vivo inhibited bleomycin-induced lung fibrosis. In vitro, GDF15 has no effect on fibroblast proliferation but does promote fibroblast differentiation to myofibroblasts and decreases fibroblast migration in collagen gel. Our in vitro data also suggest that GDF15 is involved in cell senescence, but its impact might differ depending on the cell type, as discussed further below.

The pulmonary ECM determines not only the tissue architecture, but it also provides mechanical stability and elastic recoil, which are essential for physiological lung function. It has been well established that injury and fibrosis profoundly alter the composition of ECM and matrix-associated proteins in the lung (27). Our data on proteomic analysis of decellularized ECM confirm that the IPF lung–derived matrix has altered composition. Existing evidence indicates that these changes are not only end results of fibrotic remodeling, but they are active participants in driving disease progression (28). It has been reported previously that decellularized IPF lung matrices promote fibroblast expression of αSMA (29). Furthermore, the decellularized IPF lung matrices initiate profibrotic ECM gene expression via suppression of miR-29 (30). We have shown that the decellularized matrix from lung tissue directly activates healthy normal lung fibroblasts, with ECM isolated from lung samples of patients with IPF eliciting a more profound profibrotic response. The IPF matrix markedly drives the expression of αSMA, indicative of myofibroblast differentiation.

The mechanical properties of the ECM, such as stiffness, also greatly influence the function of cells resident in the matrix, promoting fibroblast activation to a contractile and proliferative myofibroblast and, in turn, the synthesis of its own matrix components (31). This altered lung environment can further amplify cellular activation through matrix stiffness and contractile force-dependent effects on TGF-β activation, generating robust mechanical signaling to sustain fibrosis and preventing appropriate repair processes. In this context, one of the most relevant cytokines that should be considered as a major factor driving profibrotic response in our in vitro 3D system is TGF-β1 (32). A strong line of evidence shows that TGF-β–dependent signaling and TGF-β–induced events, such as epithelial-mesenchymal transition and FMT, are major players in generation of profibrotic environment. However, the level of TGF-β1 was not increased in IPF-derived matrices, similarly to what was observed by others (29). It can be explained by spatial- and time-dependent expression pattern of TGF-β, and/or it can be due to the loss of the free cytokine during the ECM decellularization process. On the other hand, profibrotic function of TGF-β depends on its activation status in ECM, which is tightly regulated and might not require upregulation of TGF-β expression (33).

We confirmed that GDF15 expression was elevated in IPF lung, while it was almost undetectable in healthy lung, and its major sources are alveolar epithelial cells and macrophages, as shown previously by others in bleomycin-treated murine lungs by IHC (23, 34) and in human IPF lungs by scRNA-Seq data sets (35).

A recent study determined that GDF15 levels correlate with age and with the extent of interstitial lung abnormalities and mortality in lung disease (36). Conversely, it has also been postulated that GDF15 plays a protective role in acute inflammation (37). In our study, neutralizing GDF15 in the bleomycin model resulted in reduced interstitial fibrosis as measured by collagen deposition. Collagen is the most abundant airway ECM component and is the primary determinant of mechanical airway properties. Its abnormal, excessive deposition is associated with the pathogenesis and progression of IPF (38). Lung fibrosis can also be assessed by measuring the quantity of dense damage area in the lung, which is a sum of different histomorphological changes, including collagen deposition, as well as inflammatory cell infiltration and accumulation, increase of thickness of the alveolar walls, enlarged hyperplastic ATII cells, edematous changes, abnormal alveolarization, fibrotic foci, and alveolar honeycombing, as was done in our study and by others (39). Although GDF15 in vivo neutralization does not result in a decrease of dense damage area, it leads to a decrease in fibrillar collagen deposition. This, after longer treatment, can potentially limit the extent of the dense damage area, as the reduction in collagen expression and deposition is linked directly to less severe fibrosis (40–42). Reduced collagen deposition resulting from antifibrotic treatment in the bleomycin mouse model was previously described and applied as an endpoint measurement by others (43, 44). Interestingly, a previous study reported that *Gdf15^–/–^* mice did not have reduced bleomycin-induced lung fibrosis (22). This may be due to the nature of the bleomycin model in that intrapulmonary bleomycin elicits profound acute lung inflammation (where GDF15 may be protective), followed by subsequent fibrotic changes and, in young healthy mice, resolution (where GDF15 may be detrimental). Constitutive gene–deficient animals, therefore, are not conducive to the study of pathways in fibrosis, as they are absent throughout the model, unless the gene deficiency is induced after the initial inflammatory phase. We used a neutralizing antibody to inhibit GDF15 after the initial inflammatory phase. Moreover, we used a bleomycin pump to systemically chronically deliver bleomycin, thereby avoiding the initial acute respiratory distress syndrome–like (ARDS-like) inflammation, as the pump model induces a more diffuse pathology (45, 46). The diffuse fibrosis elicited in the systemic bleomycin model is throughout the lung; therefore, biochemical quantification of hydroxyproline shows only a mild increase of its total collagen content in bleomycin-treated lungs, as the hydroxyproline signal is diluted when the whole lung samples are homogenized and processed. Hence, when assessing fibrosis in this model, we looked at multiple endpoints, including hydroxyproline, gene expression, and IHC, to allow for appropriate interpretation.

Although using only male mice in our study removed the sex-association aspect, there is a male dominance in IPF (47). Moreover, bleomycin promotes a stronger fibrotic response in male mice, and the dose acceptable by AstraZeneca IACUC guidelines, covering the present study to avoid any mortality, is not fibrogenic in female mice.

GDF15 was implicated in myofibroblast differentiation (48), and it has been suggested that it plays a role in cellular senescence (49), so we hypothesized that the presence of GDF15 could sustain the myofibroblast phenotype, thus preventing cellular senescence. However, we didn’t manage to confirm these results in vivo, as GDF15 neutralization didn’t show any significant impact on the gene expression of senescence markers, such as *p21* (*Cdkn1a*) and *Ccl2* (50). Nevertheless, it doesn’t exclude that GDF15 may play a role in cellular senescence. The effects of GDF15 might be cell type dependent, and GDF15 neutralization in vivo might not be sufficient to observe any decrease in the expression of senescence markers in the whole lung tissue; furthermore, the chosen readouts are not sensitive enough to see more moderate effects. Interestingly, in vitro, we observed opposite effects of GDF15 depending on the cell type. Our data indicate that, in the lung fibroblasts, GDF15 has a protective effect and might prevent cell senescence to some extent. In contrast, GDF15 neutralization led to a decreased senescence in the lung epithelial cells that suggest that GDF15 might be involved in promoting of epithelial cell senescence, as previously described (21), and that was also reported for endothelial cells (51). However, on the contrary to what was shown by Wu et al. (21), we did not reported any direct effect of rGDF15 on the expression of senescence markers in the lung epithelial cells.

Macrophages are considered crucial regulators of lung fibrosis and are often found in close proximity to collagen-producing myofibroblasts, where they can secrete numerous profibrotic soluble mediators (52, 53), potentially including GDF15. It has been postulated earlier that GDF15 can be directly linked to the enhanced expression of Ccl2 in the bleomycin mouse model (22) and may act as a macrophage recruitment signal through the CCR2/CCL2 axis (54). Neutralizing the CCR2 ligands in mice, Ccl2 and Ccl12, results in an attenuation of bleomycin-induced lung fibrosis (55–57). However, our study suggests that this is not directly linked to GDF15, as its neutralization did not lead to any decrease in Ccl2 gene expression in vivo. On the contrary, it has also been shown that GDF15 inhibited inflammatory cell recruitment in myocardial infarction, thus directly counteracting chemokine signaling and integrin activation (58, 59).

The impact of GDF15 on fibroblast-to-myofibroblast differentiation is more pronounced than its effect on cell senescence. As with the lung ECM–mediated increase in αSMA, GDF15 induced αSMA upregulation in fibroblasts, albeit not as potently as TGF-β1. Interestingly, GDF15-induced αSMA upregulation was mediated by ALK5, as the small molecule ALK5 inhibitor abolished the effects of GDF15. TGF-β–mediated signaling is considered a key event in the pathogenesis of IPF and its inhibition via a highly selective, ATP-competitive ALK5 inhibitor — SB525334 — attenuates pulmonary fibrosis in vivo (60, 61). GDF15, as a member of the TGF-β superfamily, can signal through the ALK5 receptor as reported previously (34, 59, 62, 63) or through the ALK1 receptor, leading to SMAD signaling activation in human airway epithelial cells (21) and in different cellular disease models (64, 65). These results are contradictory to what has been reported by others, who suggested that glial cell line–derived neurotrophic factor (GDNF) family receptor α like (GFRAL) is the only cell-surface receptor for GDF15 (12, 66–68). This was also confirmed in the setting of acute inflammation, where GDF15 plays a protective role and promotes survival (37). However, one cannot exclude that GDF15 may also signal through other receptors in different tissues. The expression of GFRAL is brain restricted, and we (data not shown) as well as others failed to show any expression of GFRAL in the lung or other organs, except testis, spleen, and thymus (23, 34). Thus, GFRAL-triggered signaling cannot explain all of GDF15-mediated effects reported by us and others in the context of lung fibrosis (23, 34), cancer progression (69, 70), or cardiac disease (65), as was also recently reviewed by Wischhusen et al. (71). The crystal structure of GDF15, forming a homodimer stabilized by an interchain disulfide bond, as all other TGF-β family members, suggests that some type of TGF-β receptor signaling could be involved or triggered by this cytokine (72). It was reported by Olsen et al. (73) that commercially available recombinant proteins are often contaminated by TGF-β, implying that the data showing positive impact of rGDF15 on TGF-β signaling should be interpreted with caution. However, in this study, we decided to use rGDF15 protein that was mammalian cell derived and TGF-β free, as no TGF-β contamination was detected by a highly sensitive Bio-Plex Pro TGF-β 3-plex immunoassay (Bio-Rad, 171W4001M) similarly to Olsen et al. (73).

Fibroblast phenotypical change occurring during specific pathophysiological conditions, such as fibrosis (and wound healing), involves αSMA production with stress-fiber–like appearance, further leading to migration, proliferation, and production of ECM components, such as collagen type 1. However, the hallmarks of the full transition into myofibroblast are the expression of β-cadherins, the formation of mature focal adhesions, decreased migration and proliferation, and increased contractility (74). rGDF15 used in our study had a strong impact on FMT, inducing αSMA and leading to decreased fibroblast migration, both directional (toward PDGF-BB) and spontaneous, suggesting that its presence leads to efficient myofibroblasts maturation. Its inhibitory effect on fibroblast migration could be linked to the increased adhesion, enhanced ECM deposition, and contractility, as it was reported previously that GDF15 induces contractility, cell adhesion, ECM proteins production, and αSMA expression in human trabecular meshwork cells (63). Similar effects were described for TGF-β1, which (after long-term incubation) was inducing colonic lamina propria fibroblasts into myofibroblasts with enhanced αSMA expression and reduced migratory potential (74). GDF15 was previously associated with increased migration of cancer cells (70, 75, 76) and endothelial cells (77), but to date, there is no existing evidence of its direct impact on fibroblast migration. It might trigger different effects, depending on the cell type and experimental conditions. Likewise, contrasting effects of TGF-β on fibroblast migration (depending on the incubation time, cell confluency, and cell migration assay used) are described in the literature (74, 78–80).

GDF15 seems to have multiple and often opposing roles in different pathologies, as it has been considered both as protective and deleterious. Its effect might be dose and time dependent and may vary based on the tissue environment and the cell type (81). Myofibroblasts are normally absent in healthy lung tissue, and their presence in lung parenchyma is deemed a hallmark of disease (1) and thought to contribute to the heightened stiffness of the tissue. Future studies neutralizing GDF15 in the complex ECM in vitro experiments would help to elucidate the contribution of GDF15 in this setting.

To summarize, we have shown for the first time to our knowledge that direct GDF15 neutralization could have a therapeutic effect in the treatment of lung fibrosis. Targeting of GDF15 signaling has already been considered in the context of other diseases, such as diabetes and cancer (82). Our data suggest that GDF15 may also contribute to the progression of lung fibrosis. However, its mechanism of action in the lung microenvironment requires further studies.

## Methods

### Patients.

Lungs from patients with IPF and healthy donors were acquired from the Department of Cardiothoracic Surgery, Sahlgrenska University Hospital, Gothenburg, Sweden.

### Animal care and bleomycin model of lung fibrosis.

Ten-week-old male C57BL/6 were purchased from Envigo RMS Inc. Three days prior to the experiment, mice were weighed and randomized into 6 groups. On day 0, mice received osmotic pumps (0.5 μL per hour for 7 days; Alzet, 1007D) containing either PBS (Thermo Fisher Scientific) or bleomycin (50 μg in total over 7 days; EMD Millipore Corp, 203401-10MG). Bleomycin-treated mice were given wet food to promote hydration. The weight of all the mice was monitored every other day. Therapeutic administration of 100 μL sterile PBS, control IgG (AstraZeneca, NIP228, 10 mg/kg), or anti-GDF15 mAb (10 mg/kg, R&D Systems, MAB957) was given i.p. starting at day 14 and repeated 3 times a week for a total of 5 injections. Animals were euthanized 2 days after the last treatment, on day 25.

### Gene expression analysis.

In the mouse model, RNA was isolated (Zymo Research Kit, R1065) from a snap-frozen and homogenized lobe from the right lung. RNA was reverse transcribed to cDNA using iScript Reverse Transcription kit (Bio-Rad), and Taqman probes (Thermo Fisher Scientific) were used to measure the quantity of transcript for *Cdkn1a/p21* (Mm04205640_g1), *Col3a1* (Mm00802300_m1), *Col1a1* (Mm00801666_g1), *Col1a2* (Mm00483888_m1), and *Ccl2* (Mm00441242_m1).

For human cells, RNA was isolated as described below and reverse transcribed to cDNA using High-Capacity cDNA Reverse Transcription Kit (Thermo Fisher Scientific). Taqman probes were used to measure the quantity of transcript for *COL3A1* (Hs00943809_m1), *COL1A1* (Hs00164004_m1), *CDKN1A/p21* (Hs00355782_m1), *GDF15* (Hs00171132_m1), and *TGFB1* (Hs00998133_m1).

Transcript levels of genes of interest were measured by quantitative PCR (qPCR) using QuantStudio 7 Flex Real-Time PCR System (Thermo Fisher Scientific) and were normalized to housekeeping gene mRNA, *18S* (Hs99999901_s1) in murine samples and *GAPDH* (Hs03929097_g1) or *HPRT* (hs02800695_m1) in human samples.

### Lung hydroxyproline assay in mouse lung samples.

Lung tissue was homogenized in PBS and Zirconium oxide beads (3 mm diameter). Each homogenate was transferred to a new tube, and hydrochloric acid (12N) was added in 1:1 dilution. The samples and standard samples were hydrolyzed at 96°C for 16 hours. Next, the samples were cooled to room temperature, and 300 μL acetonitrile and 20 μL internal standard (1.55M deuterium-labeled hydroxyproline) were added to each tube. After centrifugation (4,000*g*, 20 minutes, room temperature), 20 μL of supernatant was transferred to a 96-well plate and diluted with 180 μL 90% acetonitrile. The extracts were further diluted 100 times with 90% acetonitrile (4 μL extract to 396 μL solvent). The extracts were injected (2 μL) on a chromatography system consisted of a Sciex Exion pump and multisampler with an Aquity Amide column (2.1 × 50 mm, 1.7 μm particles), water (0.2% formic acid), and acetonitrile (0.2% formic acid) as mobile phases A and B. A gradient was applied starting at 5%–50% A in 2 minutes, followed by 1 minute at 50% and returning to initial conditions in 1 step. Reequilibrium time of 1 minute ended the gradient. Detection was made on a Sciex 6500+ QTRAP in positive mode with mass transitions 133.1 > 68.1 and 134.95 > 70.9 for hydroxyproline and internal standard, respectively. Acquisition and quantification were performed with Analyst 1.7 software (SCIEX).

### Histology and image analysis.

Formalin-fixed and paraffin-embedded lung sections were stained with H&E to assess gross morphology or MTC stains to visualize collagen deposition. For GDF15 detection in human lung samples, heat-induced epitope retrieval (HIER) was done in citrate (pH 6) buffer (Agilent Dako, S1699) at 110°C for 24 minutes. Intrinsic peroxidase was blocked by incubating sections with 1% hydrogen peroxide for 30 minutes, and endogenous biotin and streptavidin binding sites were blocked with Streptavidin/Biotin Blocking Kit SP-2002 (Vector Laboratories, sp2002). Sections were blocked in 5% goat serum and 1% BSA solution in PBS/0.05% Tween 20 (v/v) for 30 minutes; they were then incubated overnight with anti-GDF15 antibody (Sigma-Aldrich, HPA011191) diluted 1:300 in blocking solution. After rinsing with PBS/0.05% Tween 20 (v/v) buffer, sections were incubated with biotinylated anti–rabbit IgG (Vector Laboratories, BA-1000) solution (1:300) in blocking buffer for 1 hour. The signal was detected using the VECTASTAIN Elite ABC system (Vector Laboratories) and NovaRed solution (Vector Laboratories) according to the manufacturer’s instructions. Sections were then counterstained with H&E using standard protocol and mounted with permanent mounting medium (Pertex). For image analysis, the glass slides were converted to virtual slides using Aperio scanner. The digital files were analyzed using Visiopharm (VP) software (Version 2020.03.0.7330) with different VP scripts in order to exclude the big airways, blood vessels, and unspecific signals (e.g., anthracosis).

Lung sections (4 μm thick) were subjected to IHC staining for PSPC (Abcam, ab196677) and GDF15 (Sigma-Aldrich, HPA01119) performed on Ventana Discovery Ultra autostainer (Roche). Sequential sections were used for negative control (no primary antibodies). The slides were digitized using an OLYMPUS VS200 whole slide scanner, and the images were viewed and exported on OLYMPUS OlyVIA software (v3.2).

### Cells and cell culture conditions.

All cells were cultured in the presence of 1% (v/v) penicillin-streptomycin (Thermo Fisher Scientific, 15140122) at 37°C in a 5% CO_2_ humidified atmosphere. Primary NHLF (Lonza, CC-2512; donor LOT0000655197) were cultured in DMEM (Thermo Fisher Scientific, 31966) with 10% (v/v) FBS (Thermo Fisher Scientific, 12070-106) or in Fibroblast Growth Medium-2 (FGM-2) Bullet Kit (Lonza) supplemented with 10% FBS (spheroid assay). SAEC (Lonza, CC-2547, LOT494601) were cultured in Small Airway Epithelial Cell Growth Basal Medium (SABM; Lonza, CC-3119) supplemented with Small Airway Epithelial Cell Growth Medium SingleQuots Supplements and Growth Factors (SAGM; Lonza, CC-4124). TMLC expressing p800neoLUC (PAI1 promoter-luciferase; ref. 25) were cultured in DMEM (Thermo Fisher Scientific, 41966-029), containing 10% heat-inactivated FBS and 200 ng/mL G418 (Thermo Fisher Scientific).

### TGF-β1 activation assay.

Primary NHLF, cultured a in DMEM with 10% (v/v) FBS, were seeded in 96-well plates at the density of 1 × 10^4^ cells/well and allowed to adhere for 24 hours. Next, cells were washed twice with PBS and starved for 24 hours in DMEM containing 0.1% FBS. Then, after replacing the starvation medium with fresh DMEM with 0.1% FBS, the cells were treated with increasing concentrations (7.8125–500 ng/mL) of rGDF15 (Abcam, ab50077) alone or in the presence of 1.8 μM ALK5 inhibitor (SB525334, Sigma-Aldrich) or 0.6 ng/mL rTGF-β1 (Peprotech, 100-21). After 48 hours of incubation, the cells were fixed with 4% formaldehyde (VWR, 9713.1000) for 15 minutes at room temperature or collected for RNA extraction using RNeasy 96 Kit (Qiagen), according to the manufacturer protocol.

### Immunocytochemistry.

The 4% formaldehyde-fixed cells were washed 3 times with PBS and permeabilized with 0.2% Triton X-100 (Sigma-Aldrich) in PBS for 15 minutes. Next, after blocking with 5% BSA in PBS for 1 hour, the plates were incubated overnight at 4°C with primary antibodies diluted in blocking solution (5% BSA in PBS), monoclonal anti-αSMA antibody (Dako, clone 1A4, M085, 1:250 dilution) or anti-p21 antibody (Abcam, clone EPR362, Ab109520, 1:1000 dilution). Then, the plates were washed 3 times in PBS and incubated for 1 hour in the dark with secondary Alexa Fluor 594–conjugated goat IgG (Thermo Fisher Scientific) and Hoechst 33342 (Invitrogen, H3570) at 1:500 and 1:10,000 dilutions in PBS, respectively. After washing 3 times with PBS, the plates were sealed and image analysis was performed using ImageXpress Micro system (Molecular Devices). The percentage of αSMA^+^ or p21^+^ cells was calculated using MetaXpress High Content Image Analysis Software (Molecular Devices).

### Migration assay.

NHLF were embedded in collagen gel with or without rGDF15. After 6 hours, rhPDGF-BB (R&D Systems) was added to form a stable gradient. The migration of NHLF toward PDGF-BB was tracked using confocal live-imaging system Yokogawa CV7000 for 96 hours, and image analysis was done using Columbus software (PerkinElmer). The migration is expressed as average distance per cell, and the comparisons were made between GDF15-treated and untreated samples. The spontaneous migration of NHLF with and without GDF15 was also measured in the absence of PDGF-BB gradient.

### In vitro senescence assay.

NHLF or SAEC were seeded at the density of 5 × 10^3^ cells per well in 96-well plates. After 24-hour incubation, the medium was replaced with fresh complete DMEM, and the cells were treated with increasing concentrations of rGDF15 (62.5–500 ng/mL). After 1-hour incubation, a final concentration of 3 μM etoposide or the vehicle control (DMSO, 0.006% v/v) was added to the cells, and they were incubated for 24 hours. For RNA extraction using RNeasy 96 Kit (Qiagen), the cells were harvested by removing the medium and adding 100 μL of freshly prepared lysis buffer per well. The plates for immunostaining were fixed with 50 μL of 4% formaldehyde (VWR, 9713.1000) per well. After 15 minutes of incubation, the cells were washed 3 times with PBS and then sealed and stored at 4°C until staining. Prior to the SA–β-gal staining, the culture medium was discarded and the cells were washed with fresh cell culture medium. Then the cells were costained using Cellular Senescence Detection Kit–SPiDER-βGal (Dojindo, SG03-10) and Hoechst 33342 for nuclei detection. Next, the cells were directly imaged using ImageXpress Micro system, and the percentage of SA–β-gal^+^ cells was calculated using MetaXpress High Content Image Analysis Software.

### PAI1 expression in luciferase-reporter assay.

TMLC were seeded at the density of 3.2 × 10^4^ cells/well in white 96-well plates (Isoplate-96 TC PerkinElmer) in complete medium, and after 3 hours of growth (when the cells reached growing log phase), the medium was changed to stimulation medium (DMEM supplemented with 0.1% BSA) and increasing doses of TGF-β (0.078125–10 ng/mL) or rGDF15 (7.8125–1,000 ng/mL) were added. After overnight incubation, the cells were washed twice with PBS and lysed according to Pierce Firefly Luc One-Step Glow Assay Kit protocol (Thermo Fisher Scientific, 16196, lot TG265859). The luminescence was read from the top of the plate on EnSpire reader (Perkin Elmer).

### Proliferation assay.

For assessment of cell proliferation rate, 1.6 × 10^3^ NHLF were seeded in a 384-well plate in complete DMEM. The next day, the medium was replaced with DMEM containing 0.1% FBS, and the cells were serum starved for 4 hours. Then, the medium was replaced with fresh DMEM containing 0.1% FBS in control wells, and the cells were stimulated with PDGF-AB (150 ng/mL), rGDF15 (500 ng/mL), or FBS (5% in DMEM) in triplicate for each condition. The cells were live imaged with Incucyte S3 Live-Cell Analysis System (Essen BioScience). The images were automatically acquired in transmitted light every 4 hours over 52 hours using 10× magnification. Cell area was measured using Incucyte software, and cell confluency (%) was calculated for each well and time point. The cell proliferation was accessed based on cell confluency (%).

### ECM decellularization.

Decellularized lung matrix was prepared from adult peripheral human lung tissue obtained from pneumonectomy or cancer resection similarly to the methods described previously (83). Briefly, pieces of lung tissue measuring approximately 3 cm^3^ were snap frozen at –80°C. Prior to decellularization, frozen tissue was allowed to equilibrate to a temperature of –15°C, and thin tissue slices were cut using a scalpel blade. Tissue slices were subjected to 4 freeze-thaw cycles by shock freezing in liquid nitrogen and thawing in the water bath at 37°C. Then, the samples were lysed in 0.1% Triton X-100 in PBS with rotation on orbital shaker (Miltenyi MACSmix tube rotator) at 12 rpm at room temperature for 1 hour (all incubations steps were performed with rotation and at room temperature, unless stated differently). Next, the buffer was replaced with fresh 0.1 % Triton X-100 solution, and the samples were incubated overnight. The next day, after rinsing twice with PBS and 5 times with deionized H_2_O, the residual nuclei were lysed in 1M NaCl for 1.5 hours, followed by rinsing with PBS and twice with deionized water. Then, residual DNA was digested with 20 μg/mL DNase (Roche) in 4.2 mM solution of MgCl at 37°C for 1.5 hour (without rotation); then, the samples were rinsed twice with deionized H_2_O, followed by incubation for 30 minutes in PBS containing 1% solution of penicillin-streptomycin (10,000 U/mL) and 75 μg/mL amphotericin B (Thermo Fisher Scientific). After that, the buffer was replaced with fresh PBS containing 1% penicillin-streptomycin and 75 μg/mL amphotericin B, and the samples were incubated overnight. Next, the ECM pieces were air dried during 1–2 days in sealed plastic bags containing silica gel desiccant. Once dried, the matrix was stored at –80°C.

### Lung ECM spheroid assay.

The spheroid formation was performed using GravityPLUS plates (Insphero) according to manufacturer’s protocol. Decellularized dried lung ECM samples were weighed and crushed into powder (manual mortar). NHLF were thawed and cultured in complete FGM-2 (Lonza) supplemented with 10% FBS. Before the experiment, the cells were trypsinized, centrifuged (400*g*, 5 minutes, room temperature), and resuspended in a fresh SFM without additives and containing 5% FBS and 1% (v/v) penicillin-streptomycin. Next, 1 × 10^4^ cells per well were mixed with ECM powder at a final concentration of 0.75 mg/mL, and 40 μL of matrix cell suspension was transferred to each well of the GravityPLUS plates. The plates were incubated undisturbed at 37°C for following 3 days; then, the spheroids were transferred to trap plates, according to the manufacturer’s protocol. The medium was changed every second day, and the spheroids were harvested and fixed with 4% formaldehyde at days 4 and 8. Fixed spheroids (at least 8 spheroids per condition) were immunofluorescently labeled for αSMA and were analyzed using ImageXpress system.

### Sample preparation for global proteomic quantification.

Decellularized dried samples, crushed into powder and solubilized in about 120 μL 2% SDS and 50 mM triethylammonium bicarbonate (TEAB, Sigma-Aldrich) were shaken vigorously for 1 hour. Supernatants were transferred to new tubes, and total protein concentration was determined with Pierce BCA Protein Assay (Thermo Fisher Scientific). Aliquots containing 50 μg of each sample were digested with trypsin using the filter-aided sample preparation (FASP) method (84). Briefly, protein samples were reduced with 100 mM dithiothreitol at 60°C for 30 minutes, transferred to 30 kDa MWCO Pall Nanosep centrifugal filters (Pall Corporation), washed with 8M urea repeatedly, and alkylated with 10 mM methyl methane thiosulfate. Digestion was performed in 50 mM TEAB, 1% sodium deoxycholate buffer at 37°C by addition of Pierce MS grade Trypsin (Thermo Fisher Scientific) in a ratio of 1:100 relative to protein amount and incubated overnight. An additional portion of trypsin was added and incubated for another 4 hours.

Digested peptides were labeled using Tandem Mass Tag (TMT) 10-plex isobaric mass tagging reagents (Thermo Fisher Scientific) according to the manufacturer instructions. The labeled samples from each condition were combined into 3 separate TMT sets, and sodium deoxycholate was removed by acidification with 10% trifluoroacetic acid. Peptide aliquots corresponding to 500 μg total protein were fractionated into 42 fractions, with reverse-phase XBridge C18 3.5 μm, 3.0 × 150 mm column (Waters), using a gradient from 7% to 40% of solvent B over 32 minutes. Solvent A was 10 mM ammonium formate buffer at pH 10.00, and solvent B was 90% acetonitrile in 100 mM ammonium formate at pH 10.00. The fractions were concatenated into 21 fractions (1 + 22, 2 + 23, …21 + 42), dried with vacuum centrifugation (Genevac, miVac, max g-force 250, catalog DUC-23050-B00) at 42°C, and reconstituted in 3% acetonitrile and 0.1% formic acid for liquid chromatography–mass spectrometry (LC-MS) analysis.

### LC-MS/MS analysis and database search.

The fractions were analyzed on an Orbitrap Fusion Tribrid mass spectrometer interfaced with Easy-nLC1200 LC system (both Thermo Fisher Scientific). Peptides were trapped on an Acclaim Pepmap 100 C18 trap column (100 μm × 2 cm, particle size 5 μm; Thermo Fischer Scientific) and separated on an in-house packed analytical column (75 μm × 35 cm, particle size 3 μm, Reprosil-Pur C18, Dr. Maisch) using a linear gradient from 5% to 35% Solvent B over 75 minutes followed by an increase to 100% Solvent B for 5 minutes at a flow of 300 nL/min. Solvent A was 0.2% formic acid in water, and solvent B was 80% acetonitrile and 0.2% formic acid. For the TMT-labeled peptides, MS scans was performed at 120,000 resolution, the *m/z* range was 380–1200, and MS2 analysis was performed in a data-dependent process, with top speed cycle of 3 seconds for the most intense doubly or multiply charged precursor ions. Highest-intensity precursors were fragmented in MS2 by collision-induced dissociation (CID) at 30 collision energy with a maximum injection time of 70 ms. MS2 fragments were detected in the ion trap, followed by multinotch (simultaneous) isolation of the top 5 fragment ions, with a *m/z* range 400–1200, and were selected for further fragmentation (MS3) by higher-energy collision dissociation (HCD) at 65% and detection in the Orbitrap at 60,000 resolution, with a *m/z* range of 100–500. Precursors were isolated in the quadrupole with a 1.6 *m/z* isolation window and dynamic exclusion within 10 ppm for 60 seconds being used for *m/z* values already selected for fragmentation.

MS raw data files for each TMT set were merged for identification and relative quantification using Proteome Discoverer version 2.2 (Thermo Fisher Scientific). The database search was performed using the Mascot search engine v. 2.5.1 (Matrix Science) matching with the SwissProt H. sapiens database (https://www.uniprot.org/; accessed September 2017) with MS peptide tolerance of 5 ppm and fragment ion tolerance of 0.6 Da. Tryptic peptides were accepted with 0 missed cleavages; methionine oxidation was set as a variable modification; cysteine methylthiolation, TMT on lysine, and peptide N-termini were set as fixed modifications. Percolator was used for PSM validation with the strict FDR threshold of 1%. For the quantification, TMT reporter ions were identified in the MS3 HCD spectra with 3 mmu mass tolerance, and the TMT reporter intensity values for each sample were normalized within Proteome Discoverer 2.2 on the total peptide amount. Only unique peptide sequences at strict FDR threshold of 1% were taken into account for the protein quantification, and quantified proteins were filtered at 1% FDR. The raw data are available upon request. Only peptides unique for a given protein were considered for identification of the proteins, excluding those common to other isoforms or proteins of the same family. The raw data are available upon request.

### Proteomic data analysis.

Proteomics data were visualized using Qlucore Omics Explorer 3.6 software (Qlucore AB). PCA was used to visualize the data set in a 3-dimensional space, after filtering out variables with low overall variance to reduce the impact of noise, and centering and scaling the remaining variables to zero mean and unit variance. The projection score (85) was used to determine the optimal filtering threshold, retaining 3329 variables out of 5356 initially detected by LC-MS/MS analysis. Differentially expressed peptides between healthy and IPF patient–derived matrix samples were identified using the 2-group comparison function and unpaired *t* test with a *q* value cutoff at 0.05. The FC, defined as a the ratio of the mean values for all IPF ECM versus healthy donors, was calculated for each detected protein, and the data are expressed as log_2_(FC).

### Pathway analyses.

Pathway enrichment in IPF samples compared with healthy donors was generated through IPA (QIAGEN). Proteins considered statistically significantly differentially expressed (*q* < 0.05) were submitted to IPA to identify enriched canonical pathways. Data are displayed as IPA-generated –log(*P* value) and by the ratio of proteins present in each pathway.

### Statistics.

Analyses were performed using GraphPad Prism version 8.4.2 (GraphPad Software). The data are presented as mean ± SD using dot plots or box-and-whiskers plots, with the box extending from 75th and 25th percentiles, whiskers at the highest and lowest values, and the line in the middle plotted at the median value. The data distribution was assessed using normality tests available in GraphPad Prism software, such as Anderson-Darling, D’Agostino and Pearson, Shapiro-Wilk, and Kolmogorov-Smirnov. For the data that were normally distributed, 2-tailed *t* test was used for 2-group comparisons and multiple 1-way ANOVA was used for multiple-group comparisons. The nonparametric tests were applied for the data not normally distributed. Mann Whitney *U* test was used for 2-group comparisons and multiple Kruskal-Wallis test was used for multiple-group comparisons. The statistical significance threshold was set at *P* < 0.05.

### Study approval.

The use of transplanted IPF lungs was approved by the local ethics committee in Gothenburg, Sweden (Regionala Etikprövningsnämnden), Dnr 1026-15, and the use of healthy human lung tissue was approved by the local ethics committee in Lund, Sweden (Regionala Etikprövningsnämnden), Dnr 2013/253. All experiments were conducted in accordance with the Declaration of Helsinki. Written informed consent was received from participants prior to inclusion in the study. 

For mouse experiments, animals had full access to food and water and were treated in accordance with the *Guide for the Care and Use of Laboratory Animals* (National Academies Press, 2011) and under the American Association for the Accreditation of Laboratory Animal Care I accreditation. The AstraZeneca IACUC guidelines were applied in the study.

## Author contributions

AR, COS, and HS designed and performed in vitro experiments; CTC and JC designed, performed, and analyzed in vivo experiments; AP analyzed histological data; CS provided mass spectrometry analysis; SM, CW, and AR performed bioinformatical analysis of the data; RB performed and analyzed migration assay; MJT initiated the work on pulmonary matrix; and CMH, LAM, AR, and PH prepared figures and wrote the manuscript. All authors reviewed and provided input on the manuscript.

## Supplementary Material

Supplemental data

## Version 1 08/22/2022

Electronic publication

## Version 2 08/23/2022

Added H. Shluter's current address
