# Supplementary material for: Increased expression and accumulation of GDF15 in IPF extracellular matrix contribute to fibrosis
Source: JCI Insight. 2022 Aug 22;7(16):e153058. doi: 10.1172/jci.insight.153058 (PMC9462497; doi:10.1172/jci.insight.153058)
Supplement: Supplemental data [file jciinsight-7-153058-s300.pdf]

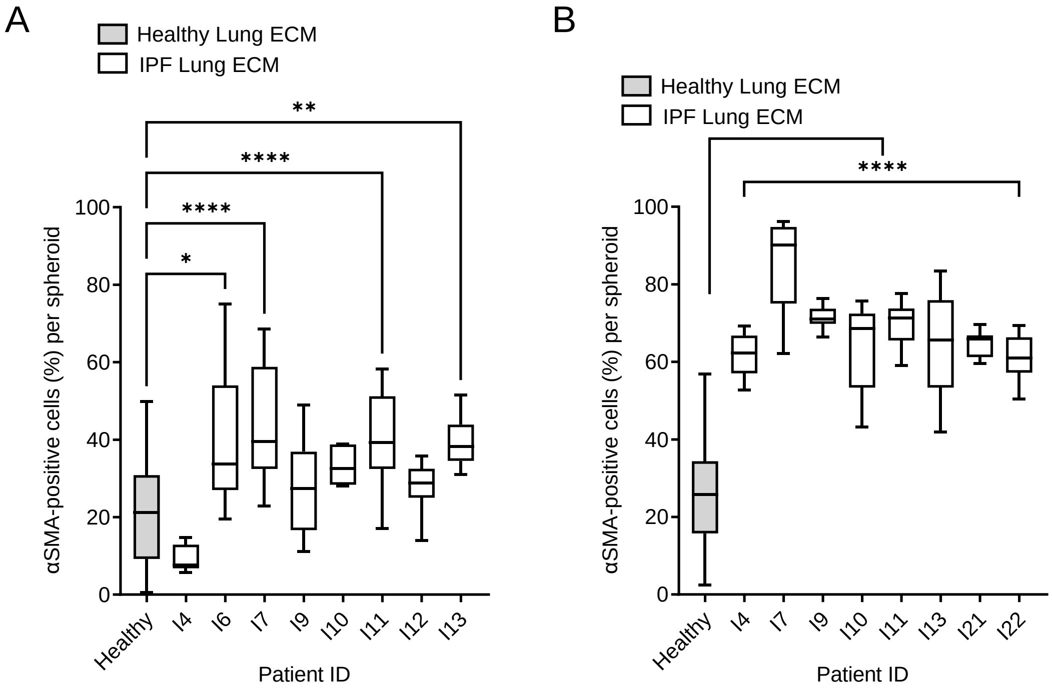

Figure S1. Diseased lung extracellular matrix promotes fibroblast to myofibroblast transition. (**A-B**) Decellularized and weight-normalized ECM generated from healthy or IPF lung tissue (n=8 donors) was co-cultured with NHLFs using 3D spheroid hanging drop system, and αSMA and nuclei were immunofluorescence labeled. Image analysis-based αSMA quantification was performed using ImageXpress Micro system and the percentage of αSMA-positive cells was calculated in each spheroid (>14 spheroids per patient, the data for the healthy group are pooled and represent the median of 8 donors) using MetaXpress High Content Image Analysis Software at day 4 (**A**) and day 8 (**B**) of culture. Data are presented using box and whiskers plots, the line in the middle is plotted at the median. Statistically analyzed with multiple Kruskal-Wallis test in comparison to healthy subjects, *p<0.05 and ****p<0.0001.

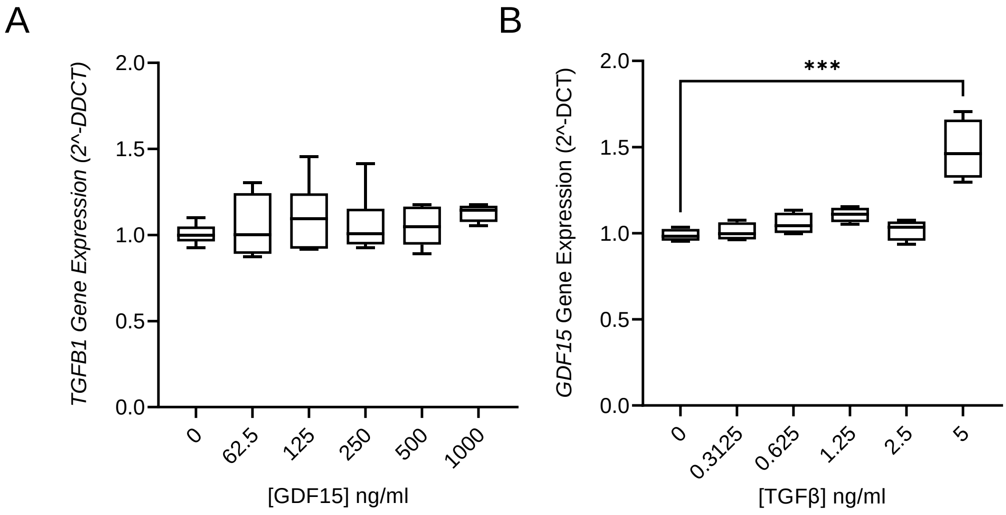

Figure S2. GDF15- or TGFβ-induced gene expression. NHLF were stimulated with recombinant GDF15 (125-1000 ng/ml) or TGFβ1 (0.3125-5 ng/ml) for 24 hours. Gene expression was measured by quantitative RT-PCR for *TGFB1* upon recombinant GDF15 stimulation (**A**) and for *GDF15* upon TGFβ1 stimulation (**B**). The expression of the genes of interest was calculated after the normalization to HPRT HKG expression and versus untreated cells, shown as 2^-DDCT (fold change) and presented using box and whiskers plots, the line in the middle plotted at the median. Statistically analyzed with nonparametric Kruskal-Wallis test, ***$p<0.001$, n=4 biological replicates.

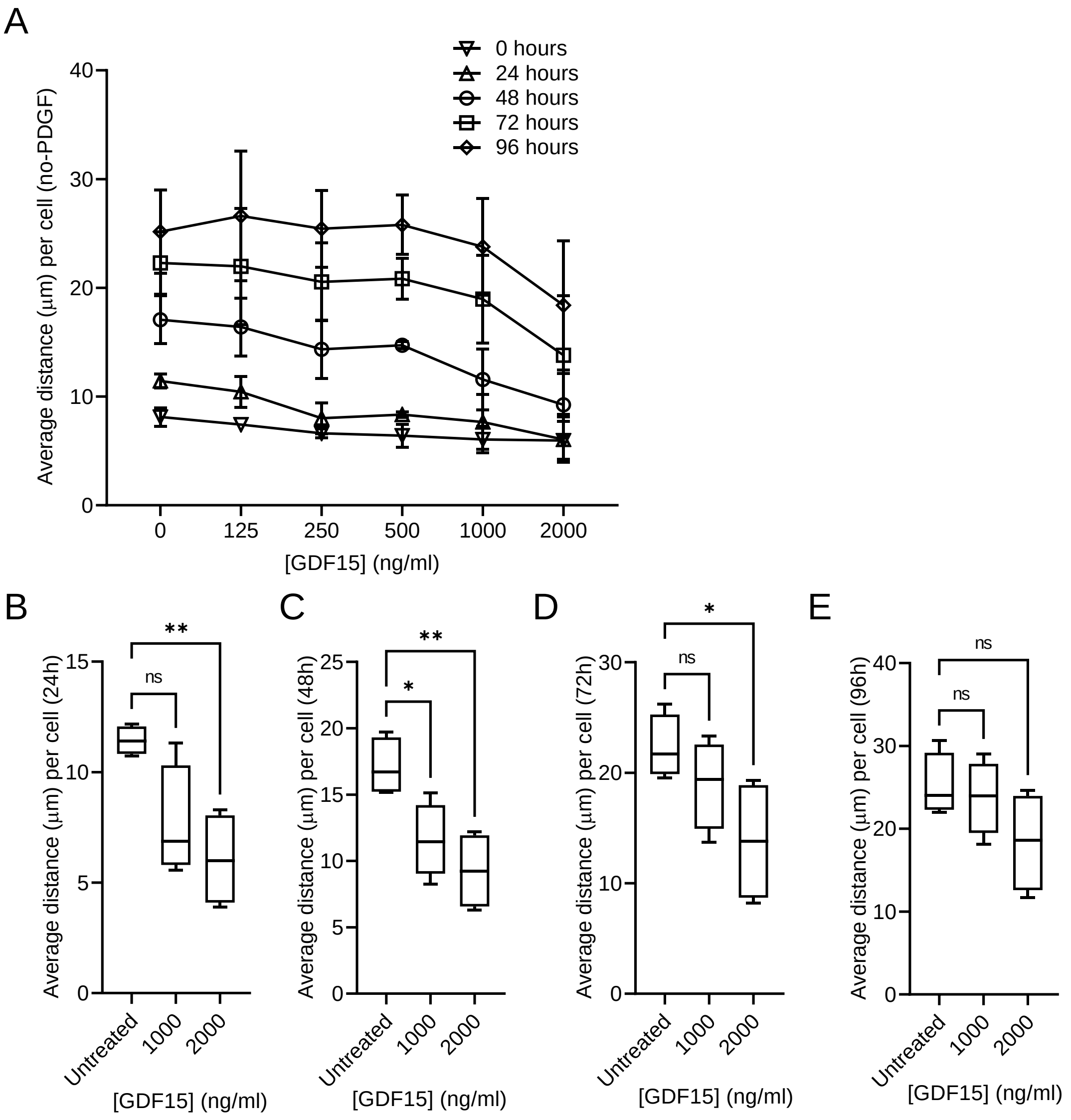

Figure S3. Stimulation with rGDF15 leads to decreased migration of NHLF. (**A**) Average migration distance of NHLF embedded in collagen gel without addition of PDGF-BB was measured with confocal live-imaging system during 24–96 hours of stimulation with rGDF15. The data are expressed as mean ± s.d. value, n=3 biological replicates. (**B-E**) The data were statistically analyzed at 24 (**B**), 48 (**C**), 72 (**D**) and 96 (**E**) hours of migration with multiple one-way ANOVA test, **p<0.01, ***p<0.001 and ****p<0.0001. Data are shown with box and whiskers plots, the line in the middle is plotted at the median.

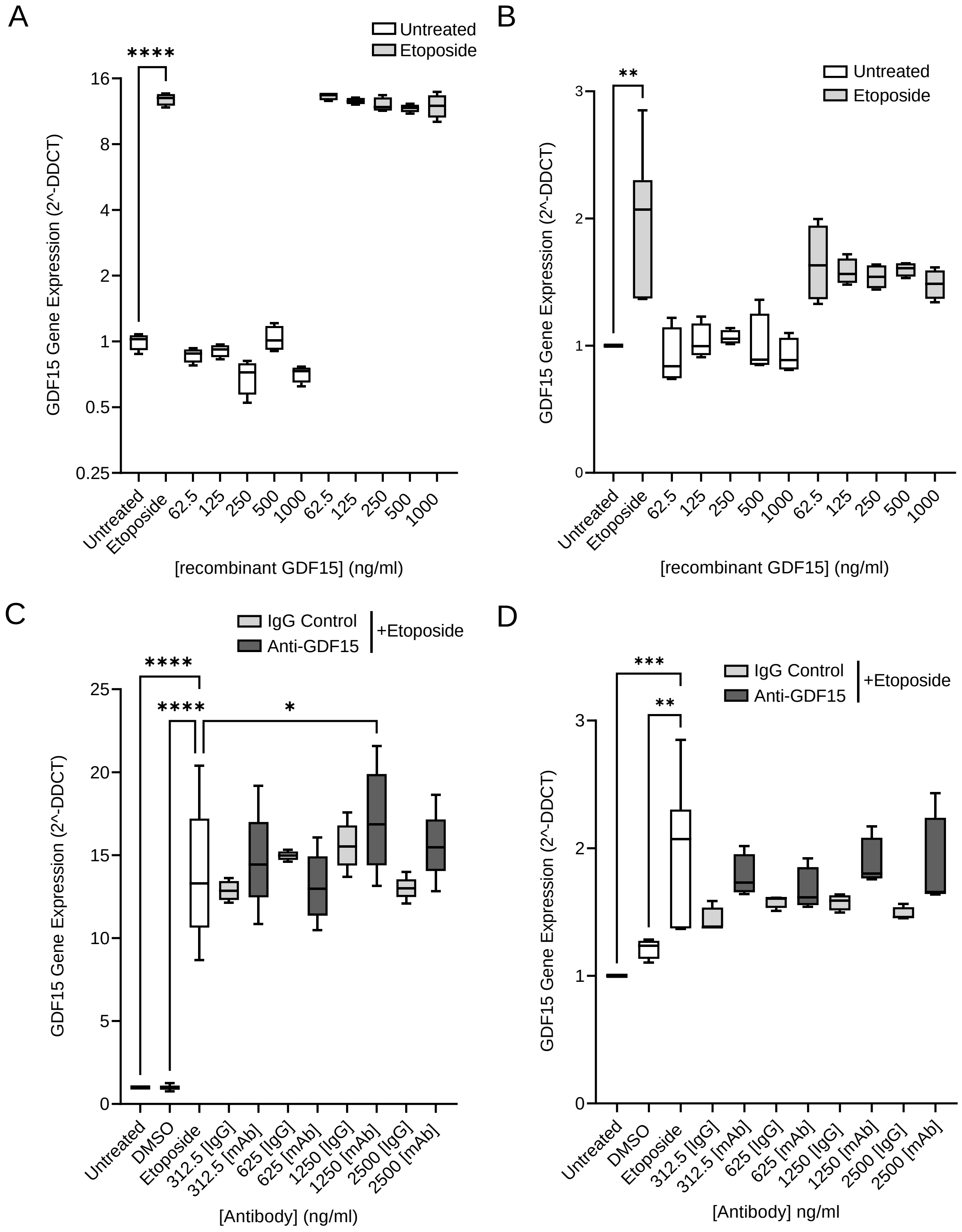

Figure S4. Impact of cellular senescence on *GDF15* expression. Cells were stimulated with etoposide (3μM) or DMSO vehicle control for 24h and pre-incubated with recombinant GDF15 (**A-B**) or with GDF15-neutralizing antibody (**C-D**). *GDF15* gene expression was measured by quantitative RT-PCR in NHLF (**A, C**) or SAEC (**B, D**). The expression of *GDF15* was calculated after the normalization to HPRT housekeeping gene expression. The data are shown as 2^-DDCT (fold change over untreated cells) and presented using box and whiskers plots, the line in the middle plotted at the median. Statistically analyzed with nonparametric Kruskal-Wallis test, *p<0.05, **p<0.01, ***p<0.001 and ****p<0.0001, n=4 biological replicates.

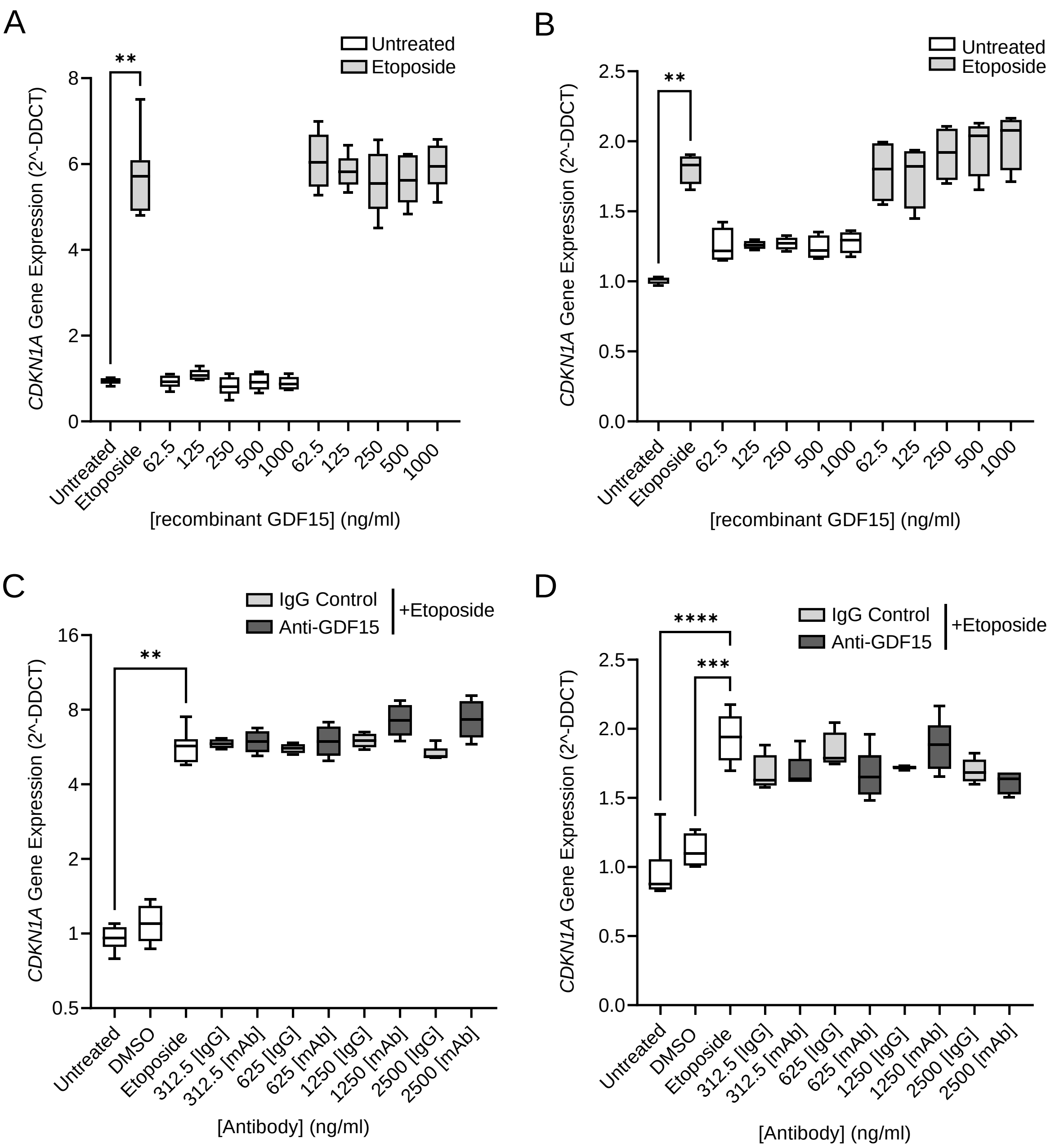

Figure S5. The effect of GDF15 on senescence-associated *p21* gene expression. Cells were treated with etoposide (3µM) or DMSO vehicle control for 24h and pre-incubated with rGDF15 (**A-B**) or with GDF15-neutralizing antibody (**C-D**). *P21 (CDKN1A)* gene expression was measured by quantitative RT-PCR in NHLF (**A, C**) or SAEC (**B, D**).The expression of the genes of interest was calculated after the normalization to HPRT housekeeping gene expression. The data are shown as 2^-DDCT (fold change over untreated cells) and presented using box and whiskers plot, the line in the middle plotted at the median. Statistically analyzed with nonparametric Kruskal-Wallis test, *p<0.05, **p<0.01, ***p<0.001 and ****p<0.0001, n=4 biological replicates.

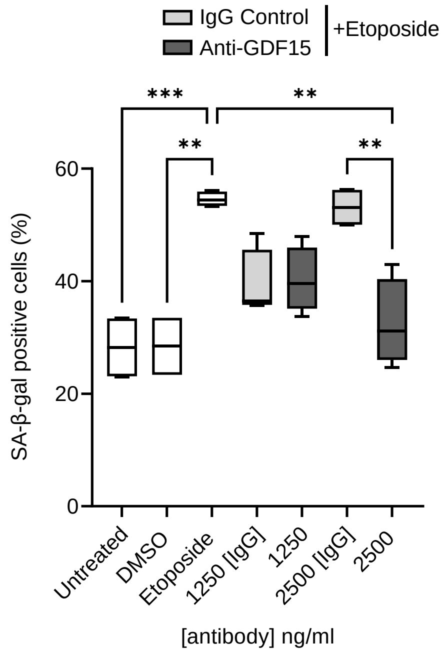

Figure S6. The effect of GDF15 neutralization on SAEC senescence. SAEC were treated with etoposide (3µM) or DMSO vehicle control for 24 hours and pre-incubated with GDF15-neutralizing antibody. Cellular SA-β-gal was detected using Cellular Senescence Detection Kit - SPiDER-βGal according to the manufacturer's protocol. Data (n=4 biological replicates) are presented using box and whiskers plot, the line in the middle plotted at the median. Statistically analyzed with multiple ANOVA analysis with Tukey's post-hoc test. $^*p<0.05$, $^{**}p<0.01$, $^{***}p<0.001$ and $^{****}p<0.0001$.

Supplemental Table 1 Information about patients used in the study.

Disease	Gender	Age range (years)	Smoking history
healthy	female	46-65	non-smoker
healthy	female	46-65	ex-smoker
healthy	male	31-45	non-smoker
healthy	male	46-65	non-smoker
healthy	male	31-45	non-smoker
healthy	male	31-45	ex-smoker
healthy	male	46-65	non-smoker
healthy	male	20-30	non-smoker
healthy	male	46-65	non-smoker
healthy	male	46-65	non-smoker
healthy	female	31-45	non-smoker
healthy	male	46-65	ex-smoker
healthy	male	46-65	ex-smoker
healthy	male	31-45	ex-smoker
healthy	female	46-65	non-smoker
healthy	female	66-85	ex-smoker
IPF	female	31-45	ex-smoker
IPF	male	66-85	ex-smoker
IPF	female	66-85	non-smoker
IPF	female	31-45	ex-smoker
IPF	female	66-85	ex-smoker
aIPF	female	46-65	ex-smoker
IPF	female	46-65	ex-smoker
IPF	female	46-65	ex-smoker
IPF	male	46-65	ex-smoker
IPF	female	46-65	ex-smoker
IPF	male	46-65	ex-smoker
IPF	male	66-85	non-smoker
IPF	female	46-65	ex-smoker
IPF	female	46-65	ex-smoker
IPF	male	66-85	ex-smoker
IPF	male	46-65	ex-smoker
IPF	male	46-65	ex-smoker
IPF	female	66-85	non-smoker
IPF	male	46-65	non-smoker

Supplemental Table 2 IPA analysis of all significantly enriched pathways (adj p value <0.05) based on differentially expressed proteins in IPF ECM compared to healthy ECM.

**Ingenuity Canonical Pathwaysa**	**-log(p-value)**	**Ratio**
FXR/RXR Activation	8.64	0.28
Acute Phase Response Signaling	8.05	0.181
LXR/RXR Activation	7.32	0.226
Hematopoiesis from Pluripotent Stem Cells	5.83	0.714
Coagulation System	5.79	0.333
Atherosclerosis Signaling	4.63	0.189
Communication between Innate and Adaptive Immune Cells	4.56	0.28
Neuroprotective Role of THOP1 in Alzheimer's Disease	4	0.2
Complement System	3.91	0.226
Intrinsic Prothrombin Activation Pathway	3.79	0.261
Systemic Lupus Erythematosus Signaling	3.35	0.125
Xenobiotic Metabolism AHR Signaling Pathway	3.12	0.171
Allograft Rejection Signaling	3.03	0.238
Ascorbate Recycling (Cytosolic)	2.84	1
Primary Immunodeficiency Signaling	2.82	0.286
Autoimmune Thyroid Disease Signaling	2.59	0.25
Gluconeogenesis I	2.59	0.25
Iron homeostasis signaling pathway	2.57	0.125
Vitamin-C Transport	2.27	0.3
IL-12 Signaling and Production in Macrophages	2.23	0.121
Osteoarthritis Pathway	2.18	0.108
Clathrin-mediated Endocytosis Signaling	2.18	0.0894
Inhibition of Matrix Metalloproteases	2.06	0.182
Extrinsic Prothrombin Activation Pathway	2.04	0.25
Aryl Hydrocarbon Receptor Signaling	2.03	0.111
Altered T Cell and B Cell Signaling in Rheumatoid Arthritis	2	0.174
Putrescine Degradation III	1.94	0.231
Lipid Antigen Presentation by CD1	1.84	0.214
Granulocyte Adhesion and Diapedesis	1.84	0.113
Xenobiotic Metabolism PXR Signaling Pathway	1.82	0.101
Tryptophan Degradation X (Mammalian, via Tryptamine)	1.76	0.2
Dopamine Degradation	1.68	0.188
Glutathione-mediated Detoxification	1.61	0.176
Pentose Phosphate Pathway	1.58	0.286
Glycolysis I	1.54	0.167
Phagosome Maturation	1.52	0.0825
LPS/IL-1 Mediated Inhibition of RXR Function	1.5	0.0875
Noradrenaline and Adrenaline Degradation	1.48	0.158
Leukotriene Biosynthesis	1.46	0.25
Choline Degradation I	1.42	1
Thyroid Hormone Biosynthesis	1.42	1
Eicosanoid Signaling	1.42	0.15
Dendritic Cell Maturation	1.36	0.0882
B Cell Development	1.36	0.222
OX40 Signaling Pathway	1.36	0.143
Serotonin Degradation	1.31	0.136
